# Characterization of Galectin Fusion Proteins with Glycoprotein Affinity Columns and Binding Assays

**DOI:** 10.3390/molecules28031054

**Published:** 2023-01-20

**Authors:** Carina Dey, Philip Palm, Lothar Elling

**Affiliations:** Laboratory for Biomaterials, Institute for Biotechnology and Helmholtz-Institute for Biomedical Engineering, RWTH Aachen University, Pauwelsstraße 20, 52074 Aachen, Germany

**Keywords:** galectin purification, glycoprotein, galectin fusion protein, Gal-1, Gal-3, Gal-8N, Gal-8C, ELISA, SNAP-tag, extracellular matrix protein

## Abstract

Galectins are β-galactosyl-binding proteins that fulfill essential physiological functions. In the biotechnological field, galectins are versatile tools, such as in the development of biomaterial coatings or the early-stage diagnosis of cancer diseases. Recently, we introduced galectin-1 (Gal-1) and galectin-3 (Gal-3) as fusion proteins of a His_6_-tag, a SNAP-tag, and a fluorescent protein. We characterized their binding in ELISA-type assays and their application in cell-surface binding. In the present study, we have constructed further fusion proteins of galectins with fluorescent protein color code. The fusion proteins of Gal-1, Gal-3, and Gal-8 were purified by affinity chromatography. For this, we have prepared glycoprotein affinity resins based on asialofetuin (ASF) and fetuin and combined this in a two-step purification with Immobilized Metal Affinity chromatography (IMAC) to get pure and active galectins. Purified galectin fractions were analyzed by size-exclusion chromatography. The binding characteristics to ASF of solely His_6_-tagged galectins and galectin fusion proteins were compared. As an example, we demonstrate a 1.6–3-fold increase in binding efficiency for HSYGal-3 (His_6_-SNAP-yellow fluorescent protein-Gal-3) compared to the HGal-3 (His_6_-Gal-3). Our results reveal an apparent higher binding efficiency for galectin SNAP-tag fusion proteins compared to His_6_-tagged galectins, which are independent of the purification mode. This is also demonstrated by the binding of galectin fusion proteins to extracellular glycoconjugates laminin, fibronectin, and collagen IV. Our results indicate the probable involvement of the SNAP-tag in apparently higher binding signals, which we discuss in this study.

## 1. Introduction

Galectins are soluble β-galactosyl binding proteins that share structural homology within their carbohydrate recognition domain (CRD). These proteins are further classified by proto-type, chimera-type, and tandem-repeat-type galectins [1]. They contribute to various physiological functions such as cell-cell and cell-matrix interactions or cell-signaling functions by binding intracellular ligands or crosslinking extracellular glycoconjugates like laminin or fibronectin [2,3,4]. Mammalian galectins are nearly expressed in all tissues [5]. In particular, an altered expression pattern of galectins can contribute to inflammation either in an attenuating or proinflammatory manner [6]. Furthermore, the galectin expression is correlated to the progress of cancer diseases [7]. In particular, Gal-1 and Gal-3 have been attributed to their role as cancer biomarkers [8,9,10,11,12].

Utilization of the functional diversity of galectins for medical and biotechnological applications of galectins has developed a considerable interest in the scientific community in the last few years. Due to the overexpression of galectins in cancer, many studies focus on the development of tailor-made galectin targeting and inhibiting agents [13,14,15]. In the medical field, galectin biosensors are developed as a diagnostic tool for the early-stage diagnosis of cancer or heart failure [16,17,18].

**Scheme 1:**
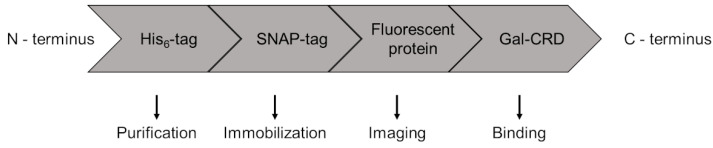
Structure of a galectin fusion protein. CRD: carbohydrate recognition domain.

The design and development of these galectin-specific tools and applications are usually preceded by detailed investigations of galectin’s carbohydrate specificities and binding affinities. During the last decades, various solid-phase, in-solution, and structural analytical methods emerged for studying galectin glycomaterials interactions [19]. In particular, solid-phase-based assays require the addition of functional domains for detection or immobilization issues. Biotinylated galectins were reported to analyze galectin binding specificities through biolayer interferometry [20]. For SPR measurements, galectins can be immobilized by fusing galectins with an AVI-tag [14]. The addition of fluorescent dyes to galectins is usually applied to detect galectin binding in microarrays [21]. In our previous studies, we introduced galectin fusion proteins consisting of a His_6_-tag for purification, a SNAP-tag for covalent immobilization, a fluorescent protein for imaging applications, and the corresponding galectin CRD (Scheme 1) [22]. Here, applications of these fusion proteins in flow cytometry, direct immobilization, and ELISA assays were demonstrated [23].

When developing galectin-specific applications, particularly in the case of fusion proteins, purity is of high importance. Human galectins are commonly acquired by recombinant expression in bacteria such as *Escherichia coli* (*E. coli*) [24]. A reliable purification method should be established when using His_6_-tagged or complex fusion proteins for in vitro studies. The main critical factors for choosing a suitable purification method are purity, protein yield, selectivity, maintenance of galectin activity, simplicity, and economic and ecological feasibility. Many strategies for galectin isolation from bacterial crude extracts have emerged in the past decades. Generally, protein characteristics like the ionic properties of galectins can be exploited by ion exchange chromatography (IEC) [25,26]. Nowadays, affinity chromatography has been established as a suitable procedure for the isolation of galectins. Here, galectins are fused to functional affinity tags either on the N- or C-terminal domain, including His_6_-tags [27,28] or Gluthatione-S-transferase (GST)-tags [24,29] which can be purified with commercially available chromatography resins. While adding affinity tags provides a selective and reproductive capture of the corresponding galectins, the co-purification of inactive galectins due to translational or misfolding issues is not excluded. The protein activity or stability can be impaired depending on the size and position of the affinity tags. In recent studies, the “Capture and Release” (CaRe) method was used to purify proteins in solution, including Gal-3 [30,31,32]. Here proteins are captured from the crude extract with target-capturing agents (TCAs). Apart from this, glycan- or glycoprotein-based purification resins represent an alternative that addresses the galectin carbohydrate specificities. The aim is to capture only active glycan-binding proteins without adding functional domains. Here, finding a universal glycan-based ligand that matches the binding characteristics of all galectins is challenging. In recent years, commercially available Lactosyl-Sepharose resins were applied to purify galectins such as Gal-1, Gal-2, Gal-3, Gal-4, and Gal-8 [27,29,33,34,35]. A Galβ1,4Fuc-decorated resin was synthesized for the selective purification of Gal-1 [36]. Since multivalency can increase the affinity of galectins, multivalent natural glycoproteins can also be used to capture galectins from the crude extract. Fetuin and ASF were utilized as natural glycoprotein ligands to purify mammalian lectins [37,38,39,40,41,42] or human antibodies [43]. Although ASF and Fetuin have been identified as suitable binders in galectin glycomaterial interactions [19,44], these glycoproteins are rarely used to purify galectins from bacterial crude extracts [33,45,46].

**Scheme 2:**
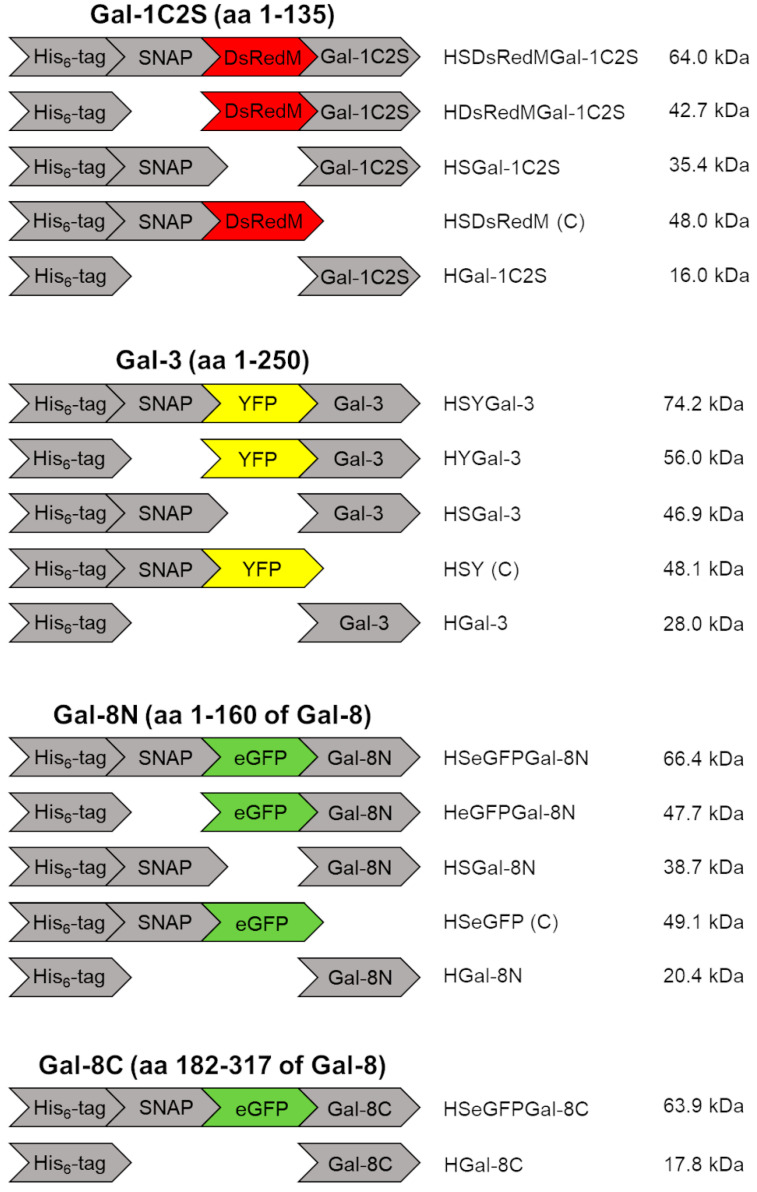
Galectin fusion proteins of this study. Fusion proteins and solely His_6_-tagged proteins of Gal-1C2S, Gal-3, Gal-8N, and Gal-8C were constructed as depicted in Scheme 1. Different fluorescent proteins were assigned to each galectin to introduce a galectin color code. Gal-1C2S, Gal-3, and Gal-8N fusion protein versions were developed without SNAP-tag, fluorescent protein, or the galectin domain (control; C). Gal: galectin; aa: amino acid; SNAP: immobilization-tag; DsRedM: monomeric red fluorescent protein; YFP: yellow fluorescent protein; eGFP, enhanced green fluorescent protein.

In this work, we aim to examine the influence of fine purification on the binding behavior of galectin fusion proteins of Gal-1, Gal-3, Gal-8N, and Gal-8C (Scheme 2). We have developed glycoprotein affinity resins based on ASF and fetuin and combined this with Immobilized Metal Affinity chromatography (IMAC) to provide pure and active galectins for our subsequent in vitro experiments.

We characterized the binding properties of different His_6_-tagged galectins and galectin fusion proteins (His_6_-SNAP-Fluorescent protein-Gal) to these multivalent glycoprotein affinity resins. In the example of Gal-3, we analyzed the influence of purification on the binding properties of His_6_-tagged Gal-3 and corresponding fusion protein constructs of Gal-3 to ASF and the extracellular matrix (ECM) glycoproteins laminin, fibronectin, and collagen IV by ELISA-type binding assays and crosslinking assays. Our results demonstrate the necessity of purification for the galectin fusion proteins and reveal the appearance of apparently high binding signals and low apparent binding constants (*K_D_*) compared to the solely His_6_-tagged galectin, which is independent of the purification mode. As we additionally observed this effect for fusion proteins of other galectin classes, we discuss the contribution of the applied functional domains of the fusion protein to this effect.

## 2. Results

### 2.1. Galectin Purification

**Figure 1:**
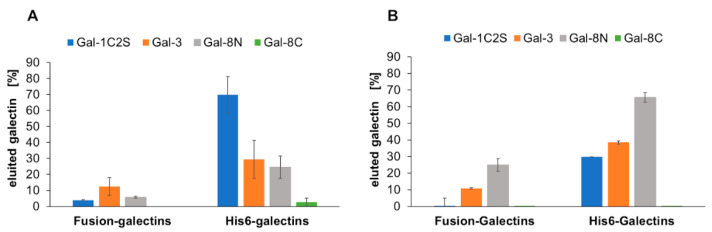
Analysis of purification for galectin fusion proteins (His_6_-SNAP-Fluorescent protein-Galectin) and His_6_-Galectins. (**A**) ASF affinity resin; (**B**) Fetuin affinity resin. 0.2 µmoL of each galectin IMAC eluate was applied to the column and eluted with PBS pH 7.5 + 300 mM lactose in a gradient mode. The amount of eluted galectin was determined by Bradford assay with bovine serum albumin as a reference. The purifications were repeated three times for each galectin.

In our studies, we aimed to establish a purification protocol that enables the fine purification of pure and active galectins for subsequent binding studies. We introduced a further affinity chromatography step after purifying His_6_-tagged galectins with IMAC. We chose the multivalent glycoproteins ASF and fetuin as binding ligands for active galectins and coupled them covalently to cyanogen bromide (CNBr)-activated Sepharose. We synthesized two different glycoprotein resins in independent coupling batches for each glycoprotein. In total, 15 mg/mL ± 0.7 mg/mL ASF and 16.4 mg/mL ± 0.9 mg/mL fetuin were coupled to Sepharose (Supplementary Materials: Table S1). In the case of fetuin-Sepharose, we additionally tested the existence of terminal sialic acid residues utilizing an enzyme-linked lectin assay (ELLA) to ensure the preservation of sialic acids during the coupling process (Supplementary Materials: Figure S1). We investigated the binding conditions regarding the incubation time of the applied sample on the column (results not shown), the buffers to be used for running the system (Figure S2), and the influence of the expression batch (Figure S3). Phosphate-buffered saline (PBS) pH 7.5 and an incubation time of 15 min were optimal conditions. We then characterized the elution of the galectin fusion proteins (His_6_-SNAP-Fluorescent protein-Galectin) and His_6_-Galectins (HGal) on the ASF-resin (Figure 1A). For this, a fixed amount (0.2 µmol) of each galectin IMAC eluate was applied to the column and was eluted with lactose in a gradient mode (Table S2, elution profiles Figures S4 and S5). We observed that almost 70% of HGal-1C2S bound to the ASF-resin, whereas only 4% of HSDsRedMGal-1C2S was eluted (Figure 1A). 30% of the applied amount of HGal-3 bound to ASF in contrast to the HSYGal-3 with 12%. The binding of Gal-8N was also observed with 25% for HGal-8N and 6% for HseGFPGal-8N. Only 3% of the applied HGal-8C was eluted, whereas no binding to the ASF-resin was detected for HSeGFPGal-8C.

The purification with fetuin-Sepharose demonstrated good binding of Gal-8N with 66% for HGal-8N and 25% for HseGFPGal-8N (Figure 1B). Furthermore, 48% of the HGal-3 and 11% of HSYGal-3 were eluted from the column with lactose. In contrast, binding to fetuin was only detected for HGal-1C2S (30%), whereas for Gal-8C, no binding was observed, and the applied protein was found in the wash fractions. In the case of Gal-3, we additionally purified the fusion protein versions HYGal-3 and HSGal-3 on the ASF-Sepharose with yields between 8% and 10%. HSY (lacking the galectin protein) was a control protein and did not bind to the glycoprotein column.

**Figure 2:**
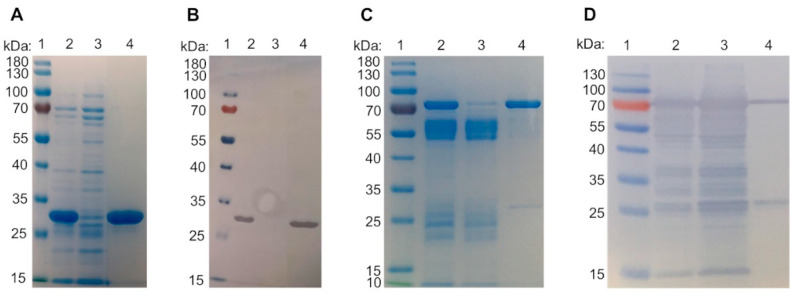
Electrophoretic analysis of Gal-3 proteins purified by IMAC and ASF affinity chromatography. (**A**) SDS-PAGE HGal-3 (28 kDa); (**B**) Western blot of HGal-3 (**C**) SDS-PAGE of HSYGal-3 (74 kDa); (**D**) Western blot of HSYGal-3. 1: Protein standard (10–180 kDa); 2: IMAC eluate; 3: Wash fractions collected in ASF affinity chromatography; 4: Eluate of ASF purification. Protein bands of SDS-PAGE were visualized by Coomassie staining. Western blot was performed with a primary anti-Gal-3-antibody and a secondary peroxidase-conjugated antibody.

The purity of the galectin proteins was analyzed by SDS-PAGE (Figure 2). The gathered IMAC eluate fractions of HGal-3 show a predominant band with a molecular weight of approximately 28 kDa and some small diffuse bands (Figure 2A, lane 2). After ASF affinity chromatography, the diffuse protein bands were separated and gathered in the wash fractions (Figure 2A, lane 3). The ASF eluate contained one dominant band at ~28 kDa (Figure 2A, lane 4), which was confirmed as HGal-3 by Western blot analysis with an anti-Gal-3-antibody (Figure 2B). The IMAC elution fractions of HSYGal-3 displayed a predominant band with a molecular weight of approximately 70 kDa. Other protein bands were observed with molecular masses ranging from ~55 kDa to ~70 kDa, and protein bands at 20 kDa, 24 kDa, and 28 kDa (Figure 2C, lane 2). However, the eluate fractions after ASF affinity chromatography gave only two bands visible at approximately 70 kDa and 28 kDa (Figure 2C, lane 4), whereas other proteins appeared in the wash fractions (Figure 2C, lane 3). These two bands were also detected in a Western blot with an anti-Gal-3 antibody (Figure 2D, lane 4). The purity of Gal-3 fusion proteins HYGal-3 and HSGal-3 after ASF affinity resin purification was also analyzed by SDS-PAGE (Figure S6). Here, we observed only one band at approx. 55 kDa and 45 kDa for each protein correspond to the molecular weight of HYGal-3 and HSGal-3, respectively. Most importantly, HSYGal-3 purified with IMAC and ASF-Sepharose showed high storage stability at 4 °C and −20 °C (Figure S7). A temperature or storage time-dependent fragmentation of HSYGal-3 was not observed.

The SDS-PAGE analysis of the His_6_-tagged and fusion proteins of Gal-1C2S and Gal-8N also displayed pure eluate fractions after IMAC and ASF affinity chromatography (Figure S8A–D, lane 8). The protein was also detected in the wash and flow-through fractions (Figure S8, lane 6 + 7). The band corresponding to the molecular weight of HGal-8C was not visible on the SDS-PAGE in all fractions gathered during IMAC and ASF-Sepharose, although binding to the ASF column was detected (Figure S8E). An additional Western blot step with an anti-His_6_-antibody confirmed the presence of the protein (Figure S9). The SDS-PAGE analysis of the His_6_-tagged galectins also revealed the functionality of the fetuin-Sepharose (Figure S10A). A fraction of HGal-1C2S has bound to fetuin and was eluted (Figure S10A, lane 1–4). HGal-3 and HGal-8N were purified well with almost no galectin protein in the flow-through and wash fractions (Figure S10A, lanes 5–8 and 9–12). As described above, the presence of HGal-8C after IMAC was detected by Western blot (Figure S9) but not after application to fetuin-Sepharose (Figure S10A, lane 13–16). The fusion proteins HSDsRedMGal-1C2S and HSeGFPGal-8C did not bind to the fetuin-resin. The galectin fusion proteins and impurities were present in the flow-through and the wash fractions (Figure S10B, lanes 1–4 and 13–16). In contrast to HGal-3 and HGal-8N, only a fraction of HSYGal-3 and HSeGFPGal-8N, respectively, bound to fetuin and were eluted. The residual fraction of the galectin fusion proteins appeared in the flow-through and wash fractions (Figure S10B, lanes 5–8 and 9–12).

We additionally analyzed the single IMAC and double purified (IMAC + ASF affinity resin) eluate fractions of HGal-3, HSYGal-3, HSGal-3, and HYGal-3 by SEC (Figure S11). After IMAC purification, the complete fusion protein HSYGal-3 eluted as three peaks (Figure S11A). Peaks 1, 2, and 3 correspond to molecular weights of 86.5 kDa, 68 kDa, and 31.7 kDa, respectively (Table 1). However, after IMAC and subsequent ASF-resin purification, HSYGal-3 eluted as one peak with a calculated molecular weight of 84.9 kDa (Figure S11B). Since the theoretical molecular weight of HSYGal-3 is 74.2 kDa, the first peaks almost corresponded to the size of the monomeric form of this fusion protein. The third peak was assigned to the fragment HS (His-SNAP = 27 kDa). The HYGal-3 IMAC fraction eluted as two peaks (Figure S11C), whereby peak 1, with a molecular weight of 47.8 kDa, matched the theoretical size of the monomeric protein. The second peak was calculated to be 31.7 kDa and matched the molecular weight of the fusion protein fragment HYFP (28.7 kDa) (Table 1). The monomeric form of HYGal-3 remained after double purification (Figure S11D). The SEC profile of HSGal-3 after IMAC purification displayed three peaks, whereas the calculated size of peak 1 almost precisely matched the monomer of HSGal-3 (Figure S11E and Table 1).

The second peak was assigned to the fragment HS (His-SNAP = 27 kDa) with a calculated molecular weight of 28.7 kDa. Peaks 2 and 3 did not appear in the SEC profile of HSGal-3 after double purification (Figure S11F). Here, only the monomeric form was detected. HGal-3 eluted as one prominent peak for both the single IMAC purification and the fine purification with IMAC and ASF-resin (Figure S11G + S11H), which fit the theoretical size of the monomeric form (Table 1). The oligomerization properties of the control protein HSY were additionally tested by SEC (Figure S12). HSY eluted as one peak with a size of 88.9 kDa corresponding to the molecular weight of the dimeric form.

### 2.2. Binding Characteristics of Gal-3 Constructs on ASF

**Figure 3:**
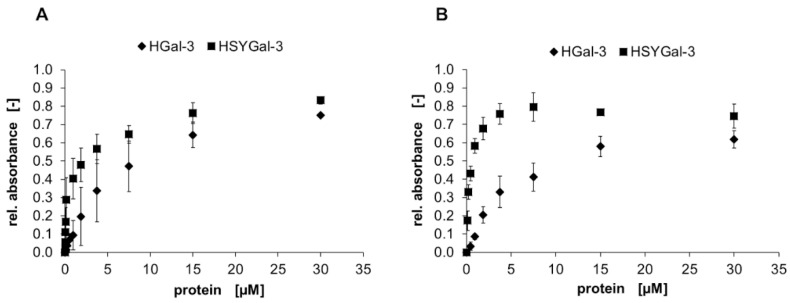
Comparison of Gal-3 binding on ASF depending on the purification mode. The binding of HGal-3 (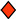) and HSYGal-3 (■) is shown for IMAC-purified (**A**) and IMAC/ASF-resin-purified (**B**) galectins. Galectin binding was detected with a peroxidase-conjugated anti-His_6_-antibody and conversion of TMB as the mean signal of three data points. Errors indicate standard deviations.

Next, we were interested in how the second purification step affects the binding of the galectin proteins in a solid-phase binding assay using ASF-coated microtiter plates. For Gal-3, we tested the binding behavior of IMAC-purified and double-purified (IMAC + ASF affinity resin) HGal-3 and Gal-3 fusion proteins. IMAC eluates of HGal-3 and fusion protein HSYGal-3 displayed an increasing binding signal depending on the galectin concentration. Maximum binding was slightly higher for HSYGal-3 (Figure 3A). The apparent equilibrium binding constant *K_D_* for HGal-3 and HSYGal-3 was calculated to be 6.9 ± 1.6 µM and 1.5 ± 0.8 µM, respectively (Table 2). The apparent binding efficiency of HGal-3 to ASF is 0.1 µM^−1^ and 0.6 µM^−1^ for HSYGal-3. Additional ASF affinity purification pronounced differences in the binding curves (Figure 3B), influencing the binding affinity and efficiencies of HGal-3 and HSY-Gal-3 (Table 2). For HGal-3, a *K_D_* value of 5.3 µM ± 1.0 µM and an apparent binding efficiency of 0.1 µM^−1^ were calculated. For HSYGal-3, the maximum binding was reached at lower protein concentrations leading to an apparent *K_D_* of 0.5 ± 0.1 µM and an apparent binding efficiency of 1.7 µM^−1^.

In summary, additional ASF affinity purification led to binding signals with more minor standard deviations. The apparent higher binding constants (*K_D_*) of HSYGal-3 compared to HGal-3 were independent of the purification mode.

**Figure 4:**
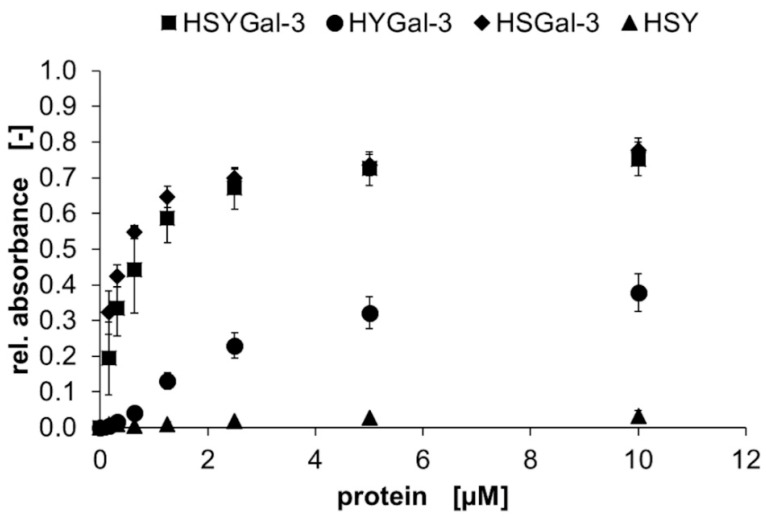
Binding of Gal-3 fusion proteins after IMAC and ASF affinity chromatography. Binding of IMAC and ASF affinity chromatography purified proteins HSYGal-3 (■), HSGal-3 (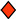), HYGal-3 (●), and HSY (▲) (control) in a solid-phase binding assay on ASF. Bound galectins were detected using a peroxidase-conjugated anti-His_6_-antibody and conversion of TMB as the mean signal of three data points. Errors indicate standard deviations.

We hypothesized that the SNAP-tag or yellow-fluorescent protein could be responsible for the differences in binding efficiencies. Both fusion partners may induce aggregates of bound galectin leading to higher binding signals. Therefore, HYGal-3 (without SNAP-tag), HSGal-3 (without YFP)*,* and HSY (control protein without galectin protein) were investigated by the solid-phase binding assay on ASF. The IMAC- and ASF-resin-purified HSGal-3 and HSYGal-3 harboring the SNAP-tag displayed higher binding signals (Figure 4) with a higher affinity to ASF compared to HYGal-3 and HGal-3 (Table 2). HSYGal-3 and HSGal-3 showed a similar progression of the binding signals (Figure 4), resulting in similar low apparent binding constants of 0.5 µM and 0.3 µM, respectively (Table 2). HSGal-3 reached the highest apparent binding efficiency of all tested galectin proteins (Table 2). A similar progression of binding signals was observed for the IMAC-purified fractions of Gal-3 fusion proteins (Figure S14). Also, apparent *K_D_* values of SNAP-tag harboring fusion proteins led to apparent higher binding efficiencies (Table 2). HSGal-3 showed the highest apparent affinity in the sub-µM level (*K_D_* = 0.3 µM) with an apparent binding efficiency of 2.7 µM^−1^. The apparent *K_D_* and binding efficiency for HSGal-3 were almost constant before and after fine purification. In contrast, the values for HSYGal-3 were improved by a factor of 3 for IMAC and ASF-resin-purified HSYGal-3 (Table 2). However, the additional purification of HYGal-3 lowered the apparent affinity and binding efficiency.

The appearance of an apparent higher binding signal for the SNAP-tag harboring fusion protein HSYGal-3 compared to HYGal-3 was further proven in a fluorescence detection-based solid-phase assay (Figure S15).

**Figure 5:**
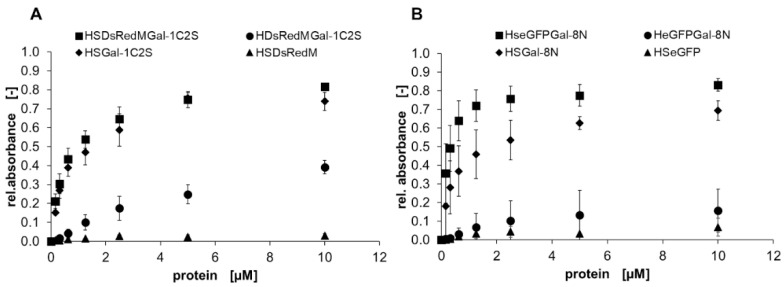
Binding of Gal-1C2S (**A**) and Gal-8N (**B**) fusion proteins after IMAC chromatography. Binding of IMAC-purified proteins HSDsRedMGal-1C2S and HSeGFPGal8N (■), HSGal-1C2S and HSGal-8N (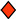), HDsRedMGal-1C2S and HeGFPGal-8N (●), HSDsRedM and HSeGFP (▲) (control) in a solid-phase binding assay on ASF. Bound galectins were detected using a peroxidase-conjugated anti-His_6_-antibody and conversion of TMB as the mean signal of three data points. Errors indicate standard deviations. The apparent binding behavior of SNAP-tagged galectins is higher compared to non-SNAP-tagged galectins.

We also investigated the binding properties of IMAC-purified His_6_-tagged and fusion proteins from proto-type galectin Gal-1C2S and the N-terminal domain (Gal-8N) of Gal-8 as a representative of the tandem-repeat galectin class (Figure 5 and Figure S16).

The SNAP-fused galectins HSDsRedMGal-1C2S and HSGal-1C2S displayed higher binding signals compared to the corresponding fusion proteins without the SNAP protein (Figure 5A). A similar curve progression revealed low binding constants of 0.6 µM and 0.8 µM for HSDsRedMGal-1CS and HSGal-1C2S, respectively, and apparent binding efficiencies in the same range (1.4 µM^−1^ for HSDsRedMGal-1C2S; 1.1 µM^−1^ for HSGal-1C2S) (Table 3). Without SNAP-fusion, HDsRedMGal-1CS showed the lowest binding signal with an up to 14 times higher apparent binding constant of 8.8 µM and a low apparent binding efficiency of 0.1 µM^−1^ (Table 3).

For Gal-8N fusion proteins, the appearance of higher binding signals for SNAP-fused proteins was also observed (Figure 5B). Here, HSeGFPGal-8N displayed the highest binding signal followed by HSGal-8N with *K_D_* values of 0.2 µM and 0.5 µM, respectively, and apparent higher binding efficiencies (Table 3). In contrast, the non-SNAP-tagged protein HeGFPGal-8N displayed a lower binding signal (Figure 5B) which resulted in a 5 to 14 times higher binding constant and a lower binding efficiency (0.1 µM^−1^) compared to the SNAP-tag harboring fusion proteins (Table 3). The *K_D_* values of fusions with fluorescent proteins of Gal-1C2S and Gal-8N (HDsRedMGal-1CS and HeGFPGal-8N) compared to the solely His_6_-tagged galectins were significantly higher by factors 18 and 8, respectively. In summary, the appearance of higher binding signals of SNAP-tagged galectins was not only limited to the chimeric protein class.

### 2.3. Binding of Gal-3 Constructs to ECM Glycoproteins

**Figure 6:**
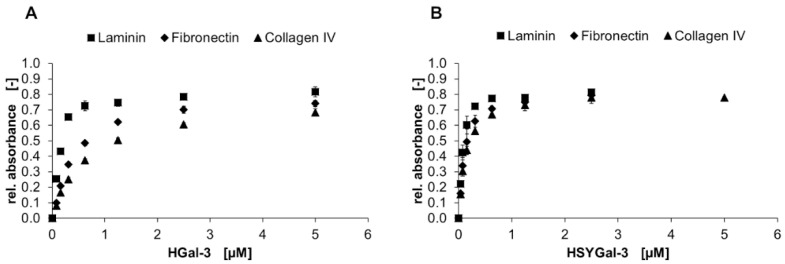
Binding of HGal-3 and HSYGal-3 on ECM glycoproteins. The binding of HGal-3 (**A**) and HSYGal-3 (**B**) on laminin (■), fibronectin (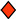), and collagen IV (▲) is shown in a solid-phase assay. Galectin binding was detected by an anti-His_6_-antibody, and conversion of TMB was measured using three data points. Error bars indicate standard deviations.

Galectin-3 plays a crucial role in cell-cell and cell-matrix interactions by crosslink formation [3,47,48,49]. For this reason, we tested the ability of His_6_-tagged galectins and galectin fusion proteins to bind to the extracellular matrix (ECM) glycoproteins laminin, fibronectin, and collagen IV in solid-phase binding assays. HGal-3 bound best to laminin, followed by fibronectin and collagen IV (Figure 6A). This was also reflected in the calculated apparent *K_D_* values showing the highest affinity of HGal-3 towards laminin (*K_D_* 0.1 µM), followed by fibronectin (*K_D_* 0.4 µM) and collagen IV (*K_D_* 0.6 µM) (Table 4). However, the binding signals of the fusion protein HSYGal-3 displayed nearly the same curve progression but here without differentiation between the single ECM glycoproteins (Figure 6B). Here, submicromolar affinities of HSYGal-3 with apparent *K_D_* values of approximately 0.1 µM towards all three extracellular matrix proteins were obtained (Table 4).

We also investigated the His_6_-tagged and fusion proteins of Gal-1C2S and Gal-8N (Figure S17). Here, we observed low binding signals of HGal-1C2S and HGal-8N for binding to fibronectin and collagen IV, which was increased when the galectins were fused to the SNAP-tag and the fluorescent protein. Furthermore, the apparent *K_D_* values of HSDsRedMGal-1C2S and HSeGFPGal-8N were also significantly reduced, being in the submicromolar range as already observed for HSYGal-3 (Table 4).

**Figure 7:**
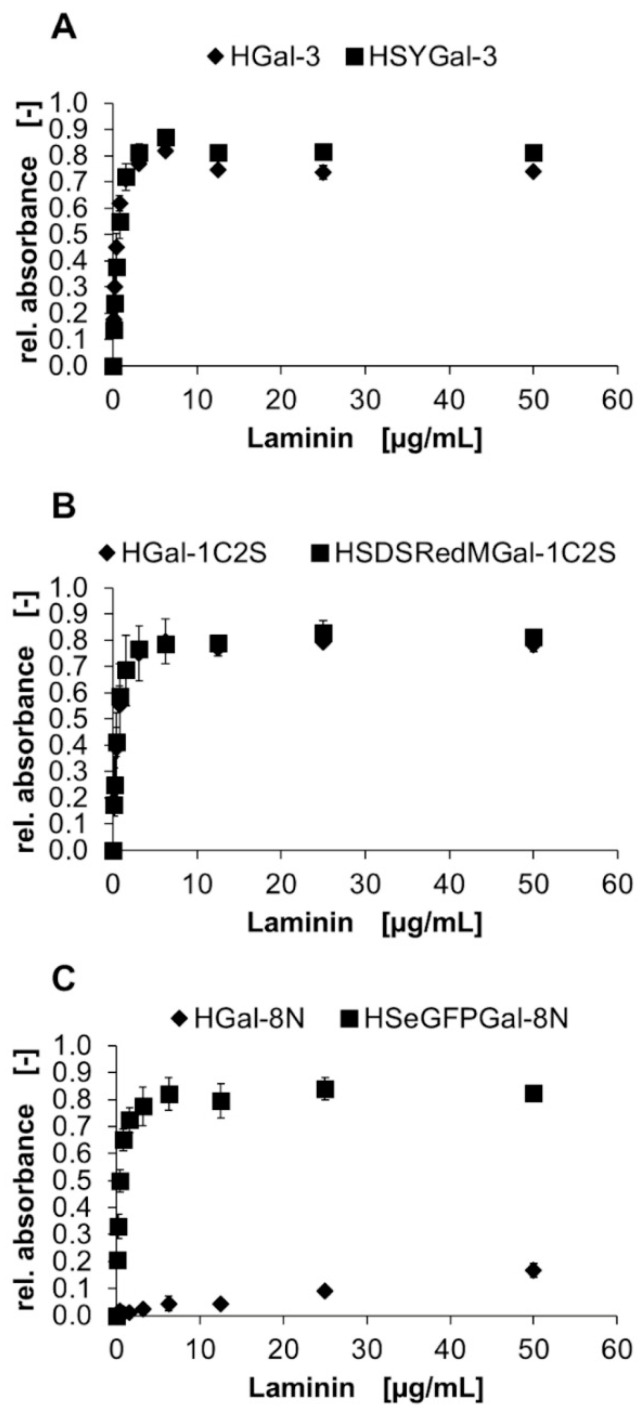
Binding of laminin to ASF-bound His_6_-tagged galectins and galectin fusion proteins. The binding of laminin is shown for ASF-bound His_6_-tagged (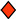) and galectin fusion proteins (■) of (**A**) Gal-1C2S (proto-type), (**B**) Gal-3 (chimera-type) and (**C**) Gal-8N (tandem-repeat-type) in a solid-phase binding assay. ECM glycoprotein crosslinking was detected by primary anti-ECM protein-antibodies and corresponding secondary peroxidase-conjugated antibodies. Conversion of TMB was measured using three data points. Error bars indicate standard deviations.

The formation of cell-cell or cell-matrix interactions requires crosslinking abilities of the mediating galectin. The ability of Gal-3 to form pentamers qualifies this protein to mediate crosslinking [45]. HGal-3 and HSYGal-3 were tested for crosslinking laminin, fibronectin, and collagen IV with a layer of ASF in a solid-phase-based assay [22]. Here, saturating amounts of galectins bound to ASF were incubated with ECM glycoproteins. We observed similar binding signals of the ECM glycoproteins to HGal-3 and HSYGal-3, with the highest binding signal for laminin, followed by fibronectin and collagen IV (Figure S18). For laminin, we obtained apparent affinities in the nM range (Table S3). Fibronectin showed higher apparent *K_D_* values in the low µM range, with 5.1 µM for HGal-3 and 10.0 µM for HSYGal-3. In contrast, the apparent binding constants for collagen IV were significantly higher, with 41.4 µM and 34.7 µM compared to fibronectin and laminin. We concluded that HGal-3 and HSYGal-3 display similar crosslinking activity for the respective ECM glycoprotein with significant differences between ECM glycoproteins. In the next step, we focused on laminin and compared its binding to ASF-bound His_6_-tagged and fusion proteins of Gal-1C2S (proto-type), Gal-3 (chimera-type), and Gal-8N (tandem-repeat-type) (Figure 7 and Table 5).

As observed for HGal-3 and HSYGal-3 (Figure 7A), the binding signals of laminin were similar for HGal-1C2S and HSDsRedMGal-1C2S with a steep increase and saturation reached at approx. 3 µM laminin (Figure 7B). This resulted in similar apparent *K_D_* values in the nM range for laminin binding to HGal-1C2S and HSDsRedMGal-1C2S (Table 5). In contrast, laminin binding to HSeGFPGal-8N compared to HGal-8N was significantly different (Figure 7C). Here, we detected a substantial increase of laminin binding to HSeGFPGal-8N whereas the binding of laminin to HGal-8N was significantly lower. This difference was also reflected in the apparent binding constants (Table 5). The affinity of laminin to bind ASF-immobilized HGal-8N was more than 230 times lower (70 µM) compared to the fusion protein HSeGFPGal-8N (0.3 µM).

## 3. Discussions

### 3.1. Galectin Purification on Glycoprotein Columns

Apart from the recombinant production of galectins, the isolation of recombinant-produced galectins from the crude extract is a crucial step for implementing in-vitro binding studies. Our studies used a two-step purification step consisting of two successive affinity purifications, including IMAC and glycoprotein affinity resin. IMAC is a well-established, commercially available purification system often used to isolate recombinant galectins [28]. We captured mg amounts of galectins from the bacterial crude extract, which is in good accordance with previous results [15,22,23,50]. Since IMAC takes advantage of the strong affinity of metal ions to histidine-peptides but does not separate active from non-active galectins, we considered using IMAC as a preselected step to get rid of the central part of the bacterial host proteome. In former studies, we used commercially available lactose-agarose resins as the second chromatography step to purify Gal-3 and Gal-3 fusion proteins [22,23]. Here, pressure issues were probably caused by the agarose resin and hampered sample application and general handling. Apart from this, the affinity of galectins towards lactose is weak [51], and we could not purify all galectins from our library with this ligand. Furthermore, the Glucose moiety from lactose is presented in open ring conformation due to the coupling chemistry to the crosslinked agarose and may also disturb the galectins’ binding. In recent years, the multivalent presentation of ligands became crucial for increasing the galectin affinities to glycan ligands [14,15,50]. Since ASF and fetuin were identified as suitable multivalent binders for galectins [19,44], we established glycoprotein-decorated affinity resins based on these natural multivalent glycoproteins. We coupled ASF and fetuin to CNBr-activated Sepharose^TM^ 4 Fast Flow with 15 mg/mL ± 0.7 mg/mL for asialofetuin and 16.4 mg/mL ± 0.9 mg/mL for fetuin, respectively (Table S1). These coupling results match the manufacturers’ information related to the coupling of α-chymotrypsinogen to CNBr-activated Sepharose (Affinity Chromatography Handbook Vol. 3: Specific Groups of Biomolecules, Cytiva). Apart from *N*-hydroxysuccinimide (NHS)- or epoxy-activated Sepharose resins, the CNBr-activated Sepharose represents a suitable pre-activated chromatography medium to couple glycoproteins by lysine residues of the protein side chains [52].

We characterized our galectin libraries’ binding properties with these glycoprotein resins, including His_6_-tagged and fusion proteins of Gal-1C2S, Gal-3, Gal-8N, and Gal-8C. Our results revealed that ASF was the best binder for HGal-1C2S with almost 70% eluted (Figure 1A), followed by HGal-3 with 30%. ASF is the desialylated form of the natural multivalent glycoprotein fetuin consisting of three tri-antennary galactose-presenting glycans and five to six core-1 O-glycan structures [53]. The binding of both galectins to ASF or *N*-acetyllactosamine (LacNAc)-terminated oligosaccharides is consistent with previous studies [44,54,55]. We also found that 25% of HGal-8N was bound to the column, although it prefers sialylated epitopes over galactosyl-terminated oligosaccharides. In contrast, we found the lowest binding for HGal-8C (3%), which has a higher affinity towards non-sialylated structures [56,57].

We further characterized the binding of the His_6_-tagged galectins on the fetuin-decorated resin (Figure 1B). Here, we observed that fetuin is a binder for HGal-8N (66%) which is due to the terminal sialylation of fetuin [53]. The N-glycans of fetuin are composed of two terminal α2,3–linked and one α2,6-linked sialic acid residue. The O-glycan is α2,6-sialylated at the *N*-acetylgalactosamine (GalNAc) and α2,3-sialylated at the galactose moiety. The preference of the Gal-8N-terminal domain for sialic acid residues, especially for α2,3 residues, was already described in previous studies [56,57]. In contrast, we detected no binding of the Gal-8C-terminal domain on the fetuin-resin, which reflects previous studies that Gal-8C binds preferentially non-sialylated oligosaccharides such as (poly)-LacNAc and blood group antigens [56,57,58,59,60]. We additionally detected binding of Gal-1 and Gal-3 with 30% and 48%, respectively. In former studies, it was found that both galectins bind α2,3 sialylated LacNAc epitopes [55]. Furthermore, sialic acids may have been lost through the coupling process to Sepharose, which could have contributed to the binding of HGal-1 and HGal-3. However, the maintenance of the sialic acids under coupling conditions was confirmed (Figure S1). The lack of HGal-8C binding to sialylated epitopes follows previous studies [51,56,57,58,59,60].

For the His_6_-SNAP-fluorescent protein-galectin fusion proteins, we observed lower binding to the ASF (Figure 1A) and fetuin (Figure 1B) affinity resins. HSYGal-3 bound best equally to ASF (12%) and fetuin (11%), whereas higher binding of HSeGFPGal-8N (25% binding) to fetuin was observed as seen for HGal-8N. No binding was found for HSeGFPGal-8C on both affinity resins. The fusion protein HSDsRedMGal-1C2S bound only to the ASF-resin in contrast to HGal-1C2S. An exception was the fusion protein of Gal-8N with 25% binding. We conclude that only a fraction of IMAC-purified galectin fusion proteins can bind to the affinity resins and are expressed as active-binding galectins. However, the well-known binding properties of the galectins are similarly reflected for the galectin fusion proteins.

We analyzed the collected IMAC fractions for all galectins by SDS-PAGE (Figure 2, Figures S6, S8 and S10). Fractions of solely His_6_-tagged galectins mainly displayed the mass corresponding to the molecular weight of these galectins with small impurities. Apart from this, the IMAC fractions of the galectin fusion proteins exhibited the mass of the whole fusion protein and several additional smaller bands with lower molecular weights, probably resulting from protein fragmentation.

We conclude that this fragmentation leads to lower yields in the glycoprotein resin chromatography for the galectin fusion proteins. In addition, yield fluctuations due to different galectin expression batches may also contribute to the overall yield of galectin gained after purification with glycoprotein resin (Figure S3). In the example of the fusion protein HSYGal-3, we examined the fragmentation which was visible in the IMAC eluate in more detail (Figure 2). The band at 74 kDa corresponds to the molecular size of the complete fusion protein HSYGal-3 (Figure 2C). Fragments that appeared at 55 kDa and 20 kDa correspond to the mass of His_6_-SNAP and YFP-Gal-3. It may arise from enterokinase cleavage, as described previously [22]. The bands at 24 kDa and 28 kDa can be assigned to the mass of YFP and Gal-3, respectively. Protein sequence analysis of the fusion protein regarding proteolytic cleavage pointed to a trypsin cleavage site between the last tyrosine residue of eYFP and the starting methionine residue of the Gal-3 protein (results not shown). However, the use of proteolytic inhibitors should prevent the observed fragmentation. Considering the fusion of four protein subunits, the fragmentation could be further induced by the IMAC procedure or result from translation abortion in protein translation. We further excluded that the fragmentation is caused by the storage conditions such as storage temperature and time (Figure S7).

We analyzed the purity of the His_6_-tagged galectins and galectin fusion proteins after ASF and fetuin affinity purification by SDS-PAGE (Figure 2, Figures S8 and S10). The purity was increased for all galectins eluted after glycoprotein affinity chromatography, indicating the purification process’s functionality. In the case of HGal-3 and HSYGal-3, protein fragments of different sizes first detected in the IMAC eluate appeared in the flow-through and wash fractions (Figure 2) since only active galectin-CRDs can bind to the ASF-decorated resin. For HSYGal-3, we observed two single bands in the ASF eluate with calculated molecular sizes of approximately 70 kDa and 28 kDa (Figure 2C, lane 4). These bands were also detected in the Western blot by an anti-Gal-3-antibody (Figure 2D, lane 4). Accordingly, the two bands can be assigned to the complete fusion protein HSYGal-3 and the single CRD domain of Gal-3, respectively. As described above, we assume that the Gal-3 domain was cleaved off from the complete fusion protein after IMAC purification and was subsequently purified by binding to the ASF-resin.

The increase in purity for HSYGal-3, HYGal-3, HSGal-3, and HGal-3 was additionally proven by SEC (Table 1 and Figure S11). Especially in the IMAC fractions of HSYGal-3 and HSGal-3, molecular masses corresponding to His-SNAP fragments were detected and strengthened the assumption of fusion protein cleavage between the SNAP-tag and the eYFP fluorescent protein. Furthermore, we observed a protein fraction in the washing step for some of the galectins in both the ASF-Sepharose (His_6_-tagged and fusion protein of Gal-8N and Gal-1C2S) and fetuin-Sepharose purification (HSYGal-3 and HGal-8N) (Figures S8A–D and S10B, 5a–12d). The appearance of the galectin in the wash step can probably be traced back to misfolded and inactive galectin. As we have purified several galectins with one column, the age of the column material may also contribute to the loss of active galectin in the eluate fraction. Generally, the galectin fusion proteins’ molecular weight is higher than the His_6_-tagged galectins. Therefore, the fusion proteins occupy a larger space, and thus other galectins could be prevented from accessing the glycans of the ASF. For Gal-8C proteins, we have observed meager yields during glycoprotein affinity purification (Figure 1), which we have traced back to the absence of a suitable glycan ligand, as described above. The SDS-PAGE analysis of IMAC fractions demonstrated recombinant expression of HSeGFPGal-8C (Figure S8F) and underlines our result that ASF and fetuin are unsuitable for affinity purification. The evaluation of the binding behavior of HGal-8C to the columns was hampered by a weak protein expression whose presence could only be detected by Western blot (Figures S8E and S9).

After two-step purification, we obtained low amounts of galectin, as we only applied 0.2 µmol galectin on the column to characterize the binding to the glycoproteins. Furthermore, we used only 1 mL of Sepharose-medium for purification. Higher volumes of chromatography media, for example, 5 mL instead of 1 mL, may allow the application of the whole IMAC eluate and could contribute to increased amounts of eluted protein after two-step purification.

Galectins are mainly purified in a one-step process by adding functional tags such as His_6_-tags or GST-tags [24,27,28,29]. The co-purification of inactive galectins due to misfolding or translational issues is not excluded. The direct one-step isolation of galectins with ASF matrices was also used in other studies for the affinity purification of Gal-3 [45,46]. Here, the maintenance of the column material could be impaired by glycosidases or proteases of the crude extract, which emphasizes the importance of a preceding purification step. In our study, introducing the second chromatography step enabled an efficient separation of protein fragments, especially in the case of galectin fusion proteins.

In contrast to our approach, a combination of DEAE-Sepharose and ASF-Sepharose was applied to purify Gal-1 from the bovine spleen with 58% recovery [33]. However, we could not cover the glycan binding specificities of all tested galectins with ASF and fetuin. The application of multivalent tailor-made glycans, such as neo-glycoproteins, shall enable the specific fine purification of these galectins [14,15,50,61]. Defined glycans were also used for fine purification [36]. Here, the purification of mouse Gal-1 mutants with a Galβ1-4Fuc-decorated resin was more efficient than an ASF resin. In addition, the CaRe method is a suitable alternative for the isolation of Gal-3. This procedure provides the isolation of Gal-3 by specific multivalent capturing agents without the use of solid chromatography resins [30,31,32]. Here, recombinant Gal-3 was purified from the crude extract with high purity using chondroitin sulfate A and C or bovine thyroglobulin as capturing agents and β-lactose as releasing disaccharide.

### 3.2. Binding of Galectins to ASF and ECM Glycoproteins

We investigated the binding properties of His_6_-tagged Gal-3 and Gal-3 fusion proteins in solid-phase assays with ASF-coated microtiter plates. For HGal-3, the fractions of IMAC and IMAC/ASF chromatography showed similar apparent *K_D_* values and binding efficiencies (Table 2). The apparent *K_D_* values are in good accordance with former results for HGal-3 [23]. The consistency before and after the fine purification can be traced back to the high purity of the protein after IMAC (Figure 2A, lane 4).

Compared to HGal-3, we observed lower apparent *K_D_* values for all SNAP-tag harboring Gal-3 fusion proteins (Table 2). For HSYGal-3, for example, this is due to a higher binding signal and lower standard deviations after ASF chromatography compared to IMAC-purified HSYGal-3 (Figure 3). However, by applying the second purification step, we expected a similar binding behavior of HSYGal-3 to that of HGal-3 due to the removal of the fragments that occurred after IMAC. We conclude that the apparent higher affinity of the galectin fusion proteins is independent of the purification mode and may be attributed to the fusion partners.

We then focused on the binding properties of different fusion protein versions of Gal-3 in the same binding assay to evaluate the influence of the single fusion partners SNAP-tag and fluorescent protein eYFP. Comparing the binding properties of solely IMAC-purified fusion protein HSYGal-3, HSGal-3, and HYGal-3 with HSY as control revealed the highest binding signal for the SNAP-tagged proteins HSGal-3 followed by HSYGal-3 (Figure S14). The use of IMAC and glycoprotein affinity purified fusion proteins did not affect the binding behavior of HSGal-3 but led to an increase of the binding signal for HSYGal-3 and resulted in a similar binding signal of HSGal-3 (Figure 4). We conclude those fusion proteins that involve more than three partners, like HSYGal-3 are susceptible to fragmentation which leads to apparent changes in binding behavior. On the other hand, the necessity for double purification of galectin fusion proteins is demonstrated. However, despite using double-purified fusion proteins of Gal-3, we still observed higher binding signals for SNAP-tagged galectins in contrast to HYGal-3, which we have also observed in a previous study [23]. As we use antibody-based His_6_-tag detection by default, we excluded a methodology-based issue causing this effect by detecting the galectin binding through fluorescence (Figure S15). The fluorescent protein eYFP tends to form weak dimers at high concentrations [62,63]. Therefore, the contribution of eYFP to the appearance of a higher binding signal cannot be entirely excluded. An increase in fluorescence intensity of fluorescent proteins upon labeling with SNAP-tag was reported [64], which could explain the enhanced binding signal of HSYGal-3 compared to HYGal-3 observed in the fluorescence detected assay (Figure S15). As we additionally observed a higher binding signal and lower apparent *K_D_* value for the fusion protein without fluorescent proteins (HSGal-3) (Figure 4, Table 2), we conclude that the SNAP-tag induces oligomerization of the galectin fusion proteins resulting in higher binding signals and an apparent higher affinity. To the best of our knowledge, the oligomerization ability of the SNAP-tag is not reported in the literature. The SNAP technology is based on the DNA repair protein *O*^6^-alkylguanine-DNA transferase (hAGT), which irreversibly transfers an alkynyl group to its cysteine group from *O*^6^-alkylguanine-DNA [65]. The crystal structure of the human monomeric SNAP-protein (RCSB PDB entry: 3KZY) displays a tertiary structure consisting of three sheets in an antiparallel direction and eight helical structures. Parts of the sheet, loop, and helical areas of the SNAP-protein consist of amino acid side chains with positive hydropathic score values (aa 30–41 from third sense sheet; aa 44–61 from loop region; aa 104–150 of the helical area) which indicate hydrophobic regions [66]. Therefore, it might be possible that the SNAP proteins interact via hydrophobic interactions under given conditions. SEC measurements of the control fusion protein HSY demonstrated the dimeric form of the protein (Figure S12), which underlines a possible contribution of the SNAP-protein and the fluorescent protein eYFP. However, SEC measurements of the double-purified fusion proteins only displayed the monomeric form of the galectin-3 protein and did not support our assumption (Table 1 and Figure S11). The absence of oligomeric structures is probably due to the measuring conditions during the SEC run. Here, protein dilution occurs as the sample flows through the column, which probably prevents the concentration-dependent formation of oligomers. In contrast, the galectins are concentrated in a smaller volume in the microtiter plate in the presence of a multivalent ligand such as ASF.

Binding assays with fusion protein versions of Gal-1C2S and Gal-8N also demonstrated the appearance of higher binding signals, resulting in low *K_D_* values for SNAP-tag harboring proteins (Figure 5, Table 3) and indicated that the effect is not restricted to the Gal-3 as representative of the chimera-type class. In the case of Gal-1C2S, we observed similar curve progressions for HSDsRedMGal-1C2S and HSGal-1C2S (Figure 5A). The red fluorescent protein DsRedM only occurs as a monomeric protein [67] and thus underlines the probable involvement of the SNAP-tag. However, eGFP can form weak dimers [68], similar to eYFP, which points out the likely influence on the high binding signal of HSeGFPGal-8N compared to HSGal-8N (Figure 5B). Furthermore, we observed lower affinities for fusion proteins with a fluorescent protein (HDsRedMGal-1CS and HeGFPGal-8N) compared to the His_6_-tagged galectins (HGal-1C2S and HGal-8N) (Table 3). This indicates a deterioration of the galectins’ binding properties in the fluorescent proteins’ presence. The fusion to SNAP-tag then again enhanced the binding in the assay on ASF, demonstrating the SNAP-tag’s contribution. We conclude that the SNAP-tag limits the use of the galectin fusion proteins in ELISA/ELLA-type assays with higher absolute read out and apparent lower *K_D_* values.

Considering the likely oligomerization of the fusion protein HSYGal-3 by the SNAP-tag, we compared the binding properties of Gal-3 proteins HGal-3 and HSYGal-3 to naturally occurring ECM glycoproteins laminin, fibronectin, and collagen IV (Figure 6). Galectins located in the extracellular space mediate cell-cell or cell-matrix interactions by crosslinking glycoproteins of the cell surface and ECM [3,4]. For both Gal-3 proteins, we detected binding to these glycoconjugates, which is in good agreement with former studies on Gal-3 and the tested ECM glycoproteins [22,47,48,49,69,70,71,72]. For HGal-3, the screening revealed graded affinities for the ECM proteins, with laminin as the best ligand with *K_D_* values in the submicromolar range, followed by fibronectin and collagen IV (Table 4). The high affinity of Gal-3 towards laminin is due to the high number of poly-*N*-acetyllactosamine-terminated N-glycans presented on this heterotrimeric ECM glycoprotein, which is a preferred ligand for Gal-3 [73,74,75,76]. Furthermore, Gal-3 can bind internal galactose residues [51]. On the contrary, fibronectin mainly presents sialylated N-glycans consisting of one lactosamine unit [75,77]. Although collagen IV is a major structural component of the extracellular space, it is modified only with galactosyl-hydroxylysine and glucosyl-galactosyl-hydroxylysine residues and, therefore, the least preferred ligand for Gal-3 [78,79]. The fusion protein HSYGal-3 displayed lower *K_D_* values than HGal-3 but without differentiation between the ECM proteins (Table 4). We also obtained the same effect in the ECM binding studies for Gal-1C2S and Gal-8N (Figure S17 and Table 4). Furthermore, these findings correlate with previously performed experiments with the galectin fusion proteins in the binding assays with ASF (Figure 3 and Figure 4). The probable oligomerization of the fusion proteins by the SNAP-tag under the given conditions in the microtiter plate leads to higher binding signals. Thus, using different ECM glycoproteins and the comparison to HGal-3 demonstrate that the high affinities of the fusion protein HSYGal-3 and other galectin fusion proteins do not reflect the natural affinity of the Gal-3 domain. These results further confirm our conclusion that the binding affinity of SNAP-tag galectin fusion proteins in ELISA type assays should be carefully discussed.

The ability of galectins to bind glycoproteins and simultaneously form oligomers enables the crosslinking and adhesion of different cells or cell-matrix components in the extracellular space [3,4,80]. In former studies, we demonstrated the ability of the Gal-3 fusion protein HSYGal-3 to crosslink laminin with an ASF layer by binding laminin to ASF-bound galectins [22]. Based on this, we have expanded the ECM spectrum and confirmed the crosslinking ability of HGal-3 and HSYGal-3 towards laminin, fibronectin, and collagen IV (Figure S18), which is due to the ability of Gal-3 to form higher multimers in the presence of multivalent ligands [45]. We then compared the laminin crosslinking ability of His_6_-tagged and fusion proteins of galectin representatives from different galectin classes. Here, we obtained a significant difference in binding and affinity for laminin between the His_6_-tagged and the fusion protein of Gal-8N (Figure 7C and Table 5). We conclude that chimera-type Gal-3 (Figure 7A) and proto-type Gal-1C2S (Figure 7B) have crosslinking activity and assume the oligomerization effect by the SNAP-tag is covered in the crosslinking assay due to the natural oligomerization behavior of these galectins. Gal-3 forms higher oligomers up to pentamers which are mediated by the glycine- and proline-rich N-terminal region, whereas Gal-1C2S is a non-covalent homodimeric protein [45,81,82]. The SNAP-tag may further trigger oligomerization and crosslinking ability. It is also reported that Gal-8 probably exists as a dimer through the interaction of the N-terminal domain [83]. Thus, the significant difference within the *K_D_* values of the HGal-8N and HSeGFPGal-8N (Table 5) may be traced back to the non-preference of Gal-8N for poly-LacNAc glycans and, on the other side, it may be attributed to the SNAP-tag fusion inducing aggregation on the ASF layer. We conclude that the SNAP-tag-induced oligomerization benefits the crosslinking properties of galectin fusion proteins.

## 4. Materials and Methods

### 4.1. Galectin Constructs

N-terminal His_6_-tag carrying galectins are based on the pETDuet-1 vector (Novagen), and galectin fusion proteins are based either on the pET17b (Novagen) or pET28a (Novagen) (Table S4). Restriction enzymes, Klenow-Polymerase I and DNA-Ligases were purchased from New England Biolabs (Germany). All gene sequences are codon-optimized for recombinant expression in *E*. *coli*, and cloning was proven by DNA sequencing. The constructs HGal-1C2S, HSDsRedGal-1C2S, HGal-3, HSYGal-3, HSGal-3, HYGal-3, and HSY used in this study were cloned as described elsewhere [22,23]. Shortly, for Gal-3 constructs, the human Gal-3 CRD, including the N-terminal glycine- and the proline-rich domain, was used (aa 1-250; Uniprot: P1731). For Gal-1 constructs, the C2S-mutated version of the human Gal-1 (aa 1-135; Uniprot: P09382) was chosen to enhance protein stability [84]. The fusion proteins HSeGFPGal-8N and HSeGFPGal-8C were synthesized in a two-step cloning process. Firstly, the synthetic gene for eGFP (*GenBank: U55762.1* [85]) was cloned from pUC18 into the existing vector pET17b:- SYGal-3 by *Age*I and *BsrG*I. The intermediate vector pET17b-HSeGFPGal-3 was then applied to introduce the synthetic domains of human Gal-8N (aa 1-160 of full-length Gal-8; Uniprot: O00214; Gene ID: 3964) and Gal-8C (aa 182-317 of full-length Gal-8 [86]) by *BsrG*I and *Not*I resulting in pET17b-HSeGFPGal-8N and pET17b-HSeGFPGal-8C, respectively. The control proteins HSDsRedM and HSeGFP were synthesized by removing the sequences coding for the corresponding galectin domains Gal-1C2S and Gal-8N from pET17b-HSeGFPGal-8N and pET17b-HSDsRedGal-1C2S via *BsrG*I and *Not*I. After eliminating residual overhanging DNA bases by Klenow polymerase I, blunt-end ligation was performed. For synthesizing fusion proteins without fluorescence (HSGal-1C2S, HSGal-8N), DNA sequences coding for DSRedM and eGFP were removed from pET17b-HSDsRedMGal-1C2S and pET17b-HSeGFPGal-8N by *Age*I and *BsrG*I which was followed by Klenow fill-in reaction and blunt-end ligation. Fusion proteins without SNAP-tag (HDsRedMGal-1C2S, HeGFPGal-8N) are based on pET28a. Here, synthetic sequences coding for DsRedMGal-1C2S and eGFPGal-8N were cloned from pUC18 into pET28a via restriction sites *Nde*I and *Not*I. The synthesis of His_6_-tagged proteins HGal-8N and HGal-8C was performed by cloning the synthetic genes for human Gal-8N (aa1-160 of full-length Gal-8; Uniprot: O00214; Gene ID: 3964) and Gal-8C (aa 182-317 of full-length Gal-8 [86]) from pUC18 into the pETDuet-1 vector by *Asc*I and *Not*I.

### 4.2. Expression and Purification

The expression of all galectin constructs was carried out in *E. coli* BL21 (DE3), *E. coli* Rosetta (DE3) pLysS, or *E. coli* Rosetta 2 (DE3) pLysS (Merck, Darmstadt, Germany) strains transformed by heat shock method (Table S4). For pre-cultivation, 20 mL LB medium was supplemented with 100 µg/mL ampicillin and 33 µg/mL chloramphenicol and inoculated with 10 µL of cryo-culture. The incubation was carried out in a baffled 100 mL flask overnight at 37 °C and 120 U × min^−1^. Precultures were then transferred to 1 L TB medium supplemented with corresponding antibiotics and incubated in a baffled 5 L flask at 37 °C and 80 U × min^−1^. Protein expression was induced by adding 1 mM IPTG at OD_600_ 0.6–0.8. and carried out at 25 °C and 80 U × min^−1^ for 24 h. Cells were harvested by centrifugation (7000 U × min^−1^, 30 min, 4 °C) and stored at −20 °C. For cell disruption, 5 g cells were resuspended in lysis buffer (20 mM NaH_2_PO_4_ pH 7.4, 0.5 M NaCl, 20 mM imidazole) followed by adding 1 µL Pierce Nuclease (Thermofisher, Dreieich, Germany). After sonification (15 s pulse, 60 s pause, amplitude 52%), the galectins were purified by IMAC (HisTrap^TM^, Cytiva, Dreieich, Germany) and dialyzed (MWCO 14 kDa) against PBS (50 mM NaH_2_PO_4_ pH 7.5, 150 mM NaCl) and stored at 4 °C for subsequent binding assays and purification with glycoprotein affinity columns.

### 4.3. Glycoprotein Affinity Resin and Galectin Purification

ASF- and fetuin-conjugated Sepharose-resin was prepared to introduce a second affinity purification step. The glycoproteins from fetal calf serum (Sigma-Aldrich, Taufkirchen, Germany) were coupled to CNBr-activated Sepharose 4 Fast Flow resin (Sigma-Aldrich, Taufkirchen, Germany) according to manufacturer instructions. Briefly, 2 g Sepharose resin was swelling in 1 mM ice-cold HCl pH 3.5 in a sintered glass filter (G3) connected to a vacuum pump. 112.5 mg of glycoprotein was solved in coupling buffer (0.2 M NaHCO_3_ pH 8.3, 0.5 M NaCl) and added to the swollen Sepharose. The coupling was carried out in an end-to-end mixer overnight at 4 °C. Washing steps with coupling buffer were performed to remove the unbound glycoprotein, followed by a quenching step with 1 mM ethanolamine pH 8 for two hours at room temperature to block the remaining reactive groups on the Sepharose. The glycoprotein-resin was subjected to alternating wash steps with coupling buffer (0.2 M NaHCO_3_ pH 8.3, 0.5 M NaCl) and washing buffer (0.1 M sodium acetate pH 4, 0.5 M NaCl) and finally stored in PBS/NaN_3_ (50 mM NaH_2_PO_4_ pH 7.5, 150 mM HCl, 0.002% (*m*/*v*) NaN_3_). For automatic purification with an Äkta system, the resin was filled in empty 1 mL columns (SepFast^TM^, BioToolmics, Consett, UK). The system was equilibrated with PBS (50 mM NaH_2_PO_4_ pH 7.5, 150 mM NaCl) to purify galectins with glycoprotein columns. After applying 0.2 µmol galectin solution, the unbound protein was washed out with 10 column volumes of PBS. The elution was performed in a gradient mode with PBS and 0.3 M lactose (0–0.3 M lactose, 15 min).

### 4.4. SDS-PAGE and Immunoblot

Discontinuous SDS-PAGE (4% stacking gel, 12–14% resolution gel) was used to analyze the galectin expression and purification. Protein samples (pellet, crude extract, flow-through, wash step, IMAC eluate, glycoprotein flow-through, wash, and eluate) were treated with 50 mM dithiothreitol (Carl Roth GmbH, Karlsruhe, Germany) and NuPAGE^TM^ LDS 1x sample buffer (Thermo Fisher Scientific, Dreieich, Germany) for 15 min at 95 °C. Gel electrophoresis was carried out at 200 V for 60 min, followed by a staining step with Coomassie Quick Stain (Serva Electrophoresis GmbH, Heidelberg, Germany). Western blotting was performed on PVDF membranes (Immobilon^®^-P, pore size 0.45 µm, Merck, Darmstadt, Germany) for 60 min at 30 V, followed by a blotting step with MeOH. Proteins were detected either with 100 mU × mL^−1^ of peroxidase-conjugated anti-His_6_-antibody (Roche GmbH, Mannheim, Germany) or anti-mouse/human Gal-3 (Mac-2) antibody (Biolegend, Koblenz, Germany). After washing with 0.05% (*v*/*v*) TBS-Tween, Pierce 1-step Ultra TMB solution (Thermo Fisher Scientific, Dreieich, Germany) was used for calorimetric visualization.

### 4.5. Size Exclusion Chromatography (SEC)

To investigate the oligomerization of different Gal-3 constructs, a Superdex 200 Increase 10/300 GL column (Cytiva, Dreieich, Germany) was used. The maximum pressure limit of the column was determined by Equations (1)–(3).

(1) pmax=Δp+Δpafter+Δpbefore

(2) Δp=Δpcolumn−Δpbefore

(3) Δpafter=Δptotal−Δpbefore(2)

Maximum pressure limit                                                             p_max_
Pressure limit at a maximum flow rate                                                         Δp
Pressure of the dripping column at max. flow rate (1.8 mL × min^−1^)                                          Δp_column_
Pressure with MQ at max. flow rate (not attached to the system)                                            Δp_before_
Pressure of column at intended conditions (0.75 mL × min^−1^, PBS pH 7.5)                                        Δp_before(2)_
Total system pressure                                                              Δp_total_

Linear regression was used to calculate the molecular weights of galectin samples. For this, the *K_AV_* (Equation (4)) of Blue Dextran and protein standards (High Molecular Weight and Low Molecular Weight; Cytiva, Germany) were plotted over the logarithmic molecular weights of the standard proteins (Figure S13). The elution volume of the samples was determined by applying 200 µL galectin sample (sterile filtered, 0.2 µM) to the column via loop injection at a constant flow rate of 0.75 mL x min^−1^ with PBS pH 7.5 as running buffer. The elution was monitored at 280 nm.

(4) $$K_{\mathrm{AV}}\left[-\right]=\frac{V_{e}-V_{o}}{V_{c}-V_{0}}$$

Average distribution constant                                                           K_AV_
Total volume of the column                                                            V_C_
Void volume                                                                    V_0_
Elution volume of standard protein/sample                                                     V_E_

### 4.6. Galectin Binding on ASF

Galectin binding analysis on ASF was based on established assays [22]. Shortly, 200 µL/well of 5 µg/mL ASF in Na_2_CO_3_ pH 9.6 was immobilized on adsorptive 96-well microtiter plates (Nunc Immuno Maxisorp, VWR, Langenfeld, Germany) overnight at 4 °C. After washing three times with PBS-Tween (0.05% *v*/*v*), the remaining active sites on the plates were blocked with 2% BSA (*m*/*v*) in PBS-Tween (0.05% *v*/*v*). Afterward, different galectin dilutions in PBS (0–30 µM; 50 µL/well) were incubated for one hour. The plates were further incubated with 50 µL of a 200 ng/µL peroxidase-conjugated anti-His_6_-antibody solution (Santa Cruz Biotechnology, Heidelberg, Germany). Galectin binding was detected with 50 µL/well 3,3′,5,5′-tetramethylbenzidine (TMB; Biotrend, Cologne, Germany and 50 µL/well 3 M HCl as a stopping reagent. Absorption was measured photometrically at 450 nm (BioTek Synergy^TM^ 2, Agilent Technologies, Santa Clara, CA, USA) as the mean signal of three data points. The binding signals were normalized with the highest binding signal, set to 1, followed by the subtraction of the background signal. Apparent *K_D_* values of galectins were calculated by non-linear fitting (SigmaPlot 14.0 software), assuming one-site saturation as galectin concentration for half maximum binding. The galectin binding efficiencies (µM^−1^) were calculated as the ratio of the maximum binding signal (B_max_) and the apparent *K_D_* value [15].

To directly detect HSYGal-3 and HYGal-3 binding via fluorescence (BioTek Synergy^TM^ 2, Agilent Technologies, Santa Clara, CA, USA), ASF was immobilized on black adsorptive 96-well microtiter plates, as described above. After incubation of galectins (0–20 µM in PBS) and three washing steps with PBS-Tween (0.05% *v*/*v*), the fluorescence of eYFP was measured with excitation filter λ = 485/20, emission filter λ = 528/20, and a 510 nm mirror in top position.

### 4.7. Galectin Binding on ECM Glycoproteins

His_6_-tagged galectins and galectin fusion proteins of Gal-1C2S, Gal-3, Gal-8N, and Gal-8C were screened for binding on extracellular matrix glycoconjugates laminin (from Engelbreth-Holm-Swarm sarcoma; Sigma-Aldrich, Taufkirchen, Germany), fibronectin (from human plasma; Sigma-Aldrich, Taufkirchen, Germany) and collagen IV (from human placenta; Sigma-Aldrich, Taufkirchen, Germany). 100 µL/well of 5 µg/mL glycoprotein solution (in Na_2_CO_3_ pH 9.6) was immobilized on adsorptive 96-well microtiter plates (Nunc Immuno Maxisorp, VWR, Germany) overnight at 4 °C. Afterward, the wells were washed 3 times with PBS-Tween (0.05% *v*/*v*) and blocked with 2% (*m*/*v*) BSA in PBS-Tween (0.05% *v*/*v*). After incubation with different galectin concentrations (0–10 µM; 50 µL/well) for 1 h, the galectin binding was detected photometrically (BioTek Synergy^TM^ 2, Agilent Technologies, Santa Clara, CA, USA) using a peroxidase-conjugated anti-His_6_-antibody, as described above for the binding assay on ASF.

### 4.8. Galectin-Mediated Crosslinking of Extracellular Matrix Glycoproteins

The ability to crosslink extracellular glycoconjugates of His_6_-tagged and galectin fusion proteins of Gal-1, Gal-3, and Gal-8N were tested as previously described [22]. Shortly, adsorptive 96-well microtiter plates (Nunc Immuno Maxisorp, VWR, Langenfeld, Germany) were coated overnight at 4 °C with 200 µL/well of a 5 µg/mL ASF solution (in Na_2_CO_3_ pH 9.6). The washing step with PBS-Tween (0.05% *v*/*v*) was followed by blocking with 250 µL of a 2% (*m*/*v*) BSA solution in PBS-Tween (0.05% *v*/*v*). After incubation with saturating amounts of the corresponding galectin (50 µM for Gal-1C2S, 25 µM for Gal-3; 40 µM for Gal-8N) for 1 h at room temperature, varying concentrations of laminin, fibronectin, or collagen IV (0–50 µM) were added for 1 h. Laminin crosslinking was primarily detected by an anti-mouse-laminin antibody from rabbit (Sigma-Aldrich, Taufkirchen, Germany; 1:1000 in PBS) and a peroxidase-conjugated anti-rabbit antibody from goat (Sigma-Aldrich, Taufkirchen, Germany; 1:1000 in PBS). For fibronectin crosslinking, a human anti-fibronectin antibody from rabbit (Sigma-Aldrich, Taufkirchen, Germany; 1:1000 in PBS) was combined with a peroxidase-conjugated anti-rabbit antibody from goat (Sigma-Aldrich, Taufkirchen, Germany; 1:1000 in PBS). Crosslinking of collagen IV from human placenta was detected using a primary human anti-collagen IV antibody from mouse (Sigma-Aldrich, Taufkirchen, Germany, 1:1000 in PBS) and a secondary peroxidase-conjugated anti-mouse antibody from goat (Sigma-Aldrich, Taufkirchen, Germany; 1:1000 in PBS). Detection was visualized photometrically (BioTek Synergy^TM^ 2, Agilent Technologies, Santa Clara, CA, USA) via the peroxidase reaction with TMB, as described above.

### 4.9. Enzyme-Linked Lectin Assay (ELLA)

An ELLA was performed to analyze the influence of glycoprotein coupling conditions on terminal sialic acid residues of fetuin. 200 µL of a 5 µg x mL^−1^ fetuin solution (in PBS) was immobilized on 96-well adsorptive microtiter plates (Nunc Immuno Maxisorp, VWR, Langenfeld, Germany) overnight at 4 °C, blocked with BSA, and washed with PBS-Tween (0.05% *v*/*v*), as described above. The adsorbed fetuin was then subjected to 50 µL of buffers used for Sepharose coupling (see Section 4.3) with PBS pH 7.5 as a control for one hour at room temperature. After washing three times, samples were incubated with 50 µL of 2 µg x mL^−1^ biotinylated *Maackia amurensis* lectin II (MALII, Roche, Mannheim, Germany) and *Sambucus nigra* elderberry bark lectin (EBL, Roche, Mannheim, Germany) in lectin buffer (10 mM HEPES pH 7.5, 150 mM NaCl, 0.1 mM CaCl_2_ ·2H_2_O) for one hour. Afterward, for one hour, lectin binding was detected with streptavidin-peroxidase (1:5000 in PBS). The absorption was measured with TMB as described.

## 5. Conclusions

Our previous studies introduced the galectin fusion protein HSYGal-3 as a potential tool for use in biomaterials’ coating applications or binding studies due to its imaging (fluorescence) and immobilization (SNAP-tag) functions. Based on this work, we enlarged our galectin fusion protein library and focused on purifying these galectins with glycoprotein affinity columns receiving pure and active binding galectins. As we could not purify all tested galectins with the glycoprotein affinity resin, follow-up studies may include the application of tailor-made multivalent neo-glycoproteins or glycoproteins with higher glycan valency and defined homogenous glycosylation for galectin-specific purification. In binding assays, we observed an apparent higher binding signal for the galectin fusion proteins compared to the His_6_-tagged proteins, which are independent of the purification mode and probably related to the oligomerization of the SNAP-tag. This limits the application of the SNAP-tag galectin fusion proteins in ELISA- or ELLA-based diagnostic applications. However, SNAP-tag-induced oligomerization can be exploited to crosslink ECM glycoproteins with glycoproteins or cell membrane glycoproteins. On the molecular level, a structural prediction of the galectin fusion proteins based on bioinformatic tools or circular dichroism spectroscopy may deliver more profound insights into the structural and conformational arrangement of the SNAP-tag within the fusion protein. A reconstruction of the galectin fusion proteins by exchange of the SNAP protein by other immobilization tools represents an additional alternative. Although the apparent *K_D_* values of the galectin fusion proteins should be considered carefully, there is versatile potential for applying galectin fusion proteins as tools in biomedical applications.

## Figures and Tables

**Table 1:** Calculated molecular weight M_W_ of eluted peaks of Gal-3 in size exclusion chromatography. Theoretic M_w_ is given in brackets (see calibration of SEC column in Figure S13).

	IMAC		IMAC + ASF Affinity Resin	
	Elution Peak	M_W_ (Calc.)	Elution Peak	M_W_ (Calc.)
HSYGal-3 (74.2 kDa)	1	86.5	1	84.9
	2	68	2	15.1
	3	31.7	3	1.8
HYGal-3 (56 kDa)	1	47.8	1	50.6
	2	30.3	2	2.0
HSGal-3 (46.9 kDa)	1	48.5	1	48.3
	2	28.7		
	3	6.2		
HGal-3 (28 kDa)	1	21.0	1	28.7
HSY (48.1 kDa)	1	88.9		

**Table 2:** Apparent *K_D_* values and binding efficiencies of IMAC and IMAC + ASF affinity column purified Gal-3 proteins in a solid-phase assay on ASF. Values were calculated by non-linear regression assuming one-site saturation.

Protein	Apparent *K_D_* [µM]	Apparent Binding Efficiency [µM^−1^]
IMAC		
HGal-3	6.9 ± 1.6	0.1
HSYGal-3	1.5 ± 0.8	0.6
HSGal-3	0.3 ± 0.02	2.7
HYGal-3	2.3 ± 0.4	0.3
IMAC and ASF affinity chromatography		
HGal-3	5.3 ± 1.0	0.1
HSYGal-3	0.5 ± 0.1	1.7
HSGal-3	0.3 ± 0.02	3.1
HYGal-3	4.1 ± 1.2	0.1

**Table 3:** Apparent *K_D_* values and binding efficiencies of IMAC-purified Gal-1C2S and Gal-8N His_6_-tagged and fusion proteins in a solid-phase assay on ASF. Values were calculated by non-linear regression assuming one-site saturation.

Protein	Apparent *K_D_* [µM]	Apparent Binding Efficiency [µM^−1^]
HSDsRedMGal-1C2S	0.6 ± 0.1	1.4
HSGal1-C2S	0.8 ± 0.1	1.1
HDsRedMGal-1C2S	8.8 ± 3.0	0.1
HGal-1C2S	0.5 ± 0.1	1.6
HSeGFPGal-8N	0.2 ± 0.1	4.0
HSGal-8N	0.5 ± 0.2	1.3
HeGFPGal-8N	2.9 ± 3.3	0.1
HGal-8N	0.37 ± 0.02	2.2

**Table 4:** Apparent *K_D_* values of HGal-3 and HSYGal-3 binding to ECM glycoproteins in a solid-phase binding assay. Values were calculated by non-linear regression assuming one-site saturation.

	Apparent *K_D_* [µM]		
	Laminin	Fibronectin	Collagen IV
HGal-3	0.13 ± 0.01	0.43 ± 0.02	0.64 ± 0.03
HSYGal-3	0.07 ± 0.01	0.11 ± 0.01	0.13 ± 0.01
HGal-1C2S	0.42 ± 0.02	1.97 ± 0.38	1.34 ± 0.15
HSDsRedMGal-1C2S	0.11 ± 0.02	0.11 ± 0.02	0.14 ± 0.03
HGal-8N	0.18 ± 0.02	0.52 ± 0.03	0.60 ± 0.03
HSeGFPGal-8N	0.04 ± 0.04	0.06 ± 0.01	0.08 ± 0.01

**Table 5:** Apparent *K_D_* values of laminin binding to ASF-bound His_6_-tagged and galectin fusion proteins of Gal-3, Gal-1C2S, and Gal-8N in a solid-phase binding assay. Values were calculated by non-linear regression assuming one-site saturation.

ASF-Bound Galectin	Apparent *K_D_* [µM] of Laminin
HGal-3	0.27 ± 0.03
HSYGal-3	0.45 ± 0.04
HGal-1C2S	0.38 ± 0.07
HSDsRedMGal-1C2S	0.37 ± 0.03
HGal-8N	69.5 ± 22.7
HSeGFPGal-8N	0.27 ± 0.02

## Data Availability

The data presented in this study are available in the article and in the Acknowledgments: The authors thank Niels Hoffmann, Nikol Kodra, Esther Hudina, and Nina Klos for their support in the experiments.

## Supplementary Materials

The following supporting information can be downloaded at: https://www.mdpi.com/article/10.3390/molecules28031054/s1, Figure S1: ELLA of fetuin under coupling conditions; Figure S2: Buffers for ASF affinity purification; Figure S3: Dependency on the expression batch; Figure S4–S5: Elution profiles glycoprotein affinity chromatography; Figure S6, S8–S10: SDS-PAGE analysis of galectin purifications; Figure S7: Analysis of HSYGal-3 storage stability; Figure S11–S12: SEC profiles of Gal-3 proteins; Figure S13; Calibration line for SEC; Figure S14: Binding of Gal-3 fusion proteins after IMAC; Figure S15: Fluorescence detected binding of Gal-3 fusion proteins; Figure S16: Binding of His_6_-tagged and fusion proteins of Gal-1C2S and Gal-8N; Figure S17–S18: ECM binding of galectins. Table S1: Glycoprotein coupling; Table S2: Lactose concentrations at galectin elution; Table S3: Binding constants of laminin for ASF-bound Gal-3 proteins; Table S4: *E. coli* strains used for recombinant expression of galectin constructs.
