# Supplementary material for: Characterization of Galectin Fusion Proteins with Glycoprotein Affinity Columns and Binding Assays
Source: Molecules. 2023 Jan 20;28(3):1054. doi: 10.3390/molecules28031054 (PMC9919667; doi:10.3390/molecules28031054)
Supplement: Supplementary file 1 [file molecules-28-01054-s001.zip › molecules-2141807-supplementary.pdf]

# Supporting information

## Characterization of galectin fusion proteins with glycoprotein affinity columns and binding assays

Carina Dey, Philip Palm, and Lothar Elling*
*Laboratory for Biomaterials, Institute for Biotechnology and Helmholtz-Institute for Biomedical Engineering, RWTH Aachen University, Pauwelsstraße 20, 52074 Aachen, Germany*

* Correspondence: l.elling@biotec.rwth-aachen.de; Tel.: +49 241 8028350

**Table S1.** Results of ASF and fetuin coupling to CNBr-activated Sepharose. Glycoprotein amounts were calculated by Bradford assay.

| Glycoprotein: | Amount [mg] | Unbound [mg] | Coupled [mg] | Coupled [%] | Binding capacity [mg/mL] |
|---|---|---|---|---|---|
| ASF (Batch I) | 112.5 | 26.5 | 86.0 | 76.8 | 15.7 |
| ASF (Batch II) | 112.5 | 34.0 | 78.5 | 2.0 | 14.3 |
| Fetuin (Batch I) | 112.5 | 26.1 | 86.4 | 76.4 | 17.2 |
| Fetuin (Batch II) | 112.5 | 27.1 | 85.4 | 76.0 | 15.5 |

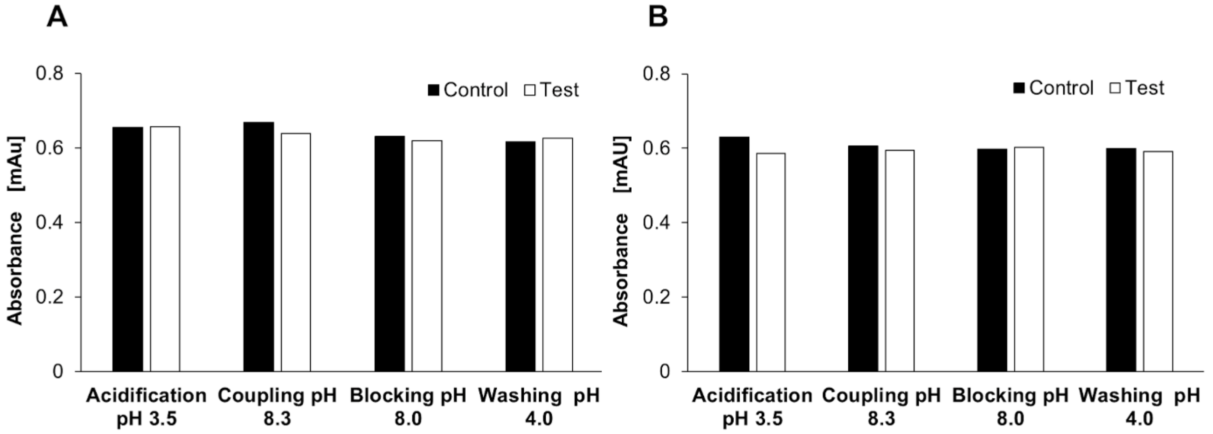


**Figure S1.** Enzyme-linked lectin assay (ELLA) of fetuin at different CNBr-activated sepharose coupling conditions.
**A:** Detection of the terminal of α2,3 sialic acids with *Maackia amurensis* lectin II (MAL II); **B:** Detection of terminal α2,6 sialic acids with *Sambucus nigra* elderberry bark lectin (EBL). Test: Treated fetuin sample with acidification solution pH 3.5, coupling buffer pH 8.0, blocking buffer pH 8.0, or washing buffer pH 4.0; Control: Untreated fetuin sample.

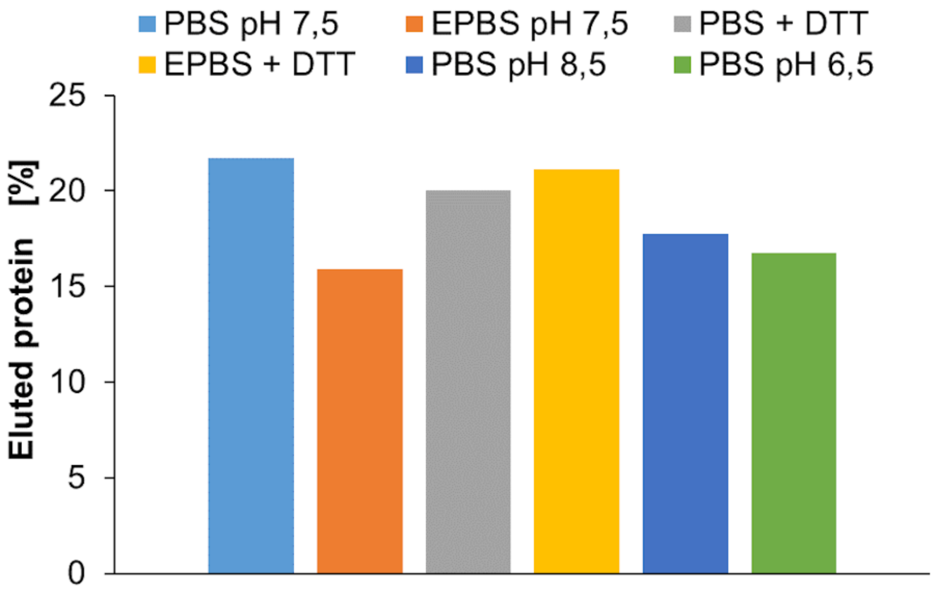


**Figure S2.** Comparison of buffers for binding HSYGal-3 on ASF-Sepharose.
3 mg of HSYGal-3 from one single expression batch was applied to the column in different buffers and eluted with lactose in a gradient mode (0-0.3 M lactose in PBS pH 7.5 within 15 min). The highest amount of eluted protein was observed for HSYGal-3 in PBS pH 7.5.

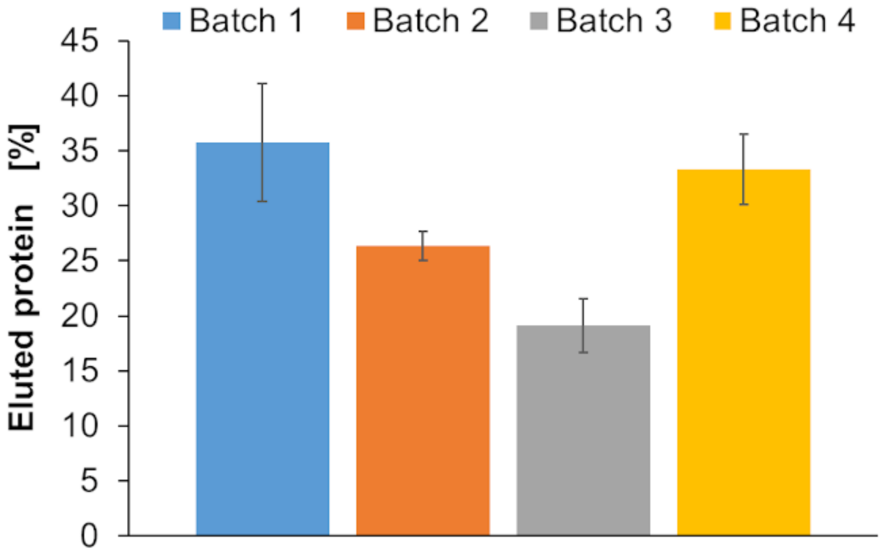


**Figure S3.** Dependency of expression batch on HSYGal-3 yield in ASF affinity chromatography.
4 mg of HSYGal-3 IMAC eluate of four different expression batches were applied to the ASF-Sepharose and eluted with lactose in a gradient mode (0-0.3 M lactose in PBS pH 7.5 within 15 min).

**Table S2.** Lactose concentrations for elution of galectins in glycoprotein affinity chromatography. Lactose concentrations were determined by gradient elution as the mean signal of two data points for ASF-Sepharose. For Fetuin-Sepharose lactose concentration was calculated once.

| | **Lactose concentration [mM]** | |
|---|---|---|
| | **ASF-Sepharose** | **Fetuin-Sepharose** |
| HGal-1C2S | 30 ± 5 | 20 |
| HSDsRedMGal-1C2S | 22 ± 2 | 27 |
| HGal-3 | 36 ± 8 | 25 |
| HSYGal-3 | 25 ± 4 | 22 |
| HGal-8N | 20 ± 0 | 32 |
| HseGFPGal-8N | 16 ± 1 | 30 |
| HGal-8C | - | - |
| HSeGFPGal-8C | 20 ± 0 | 23 |

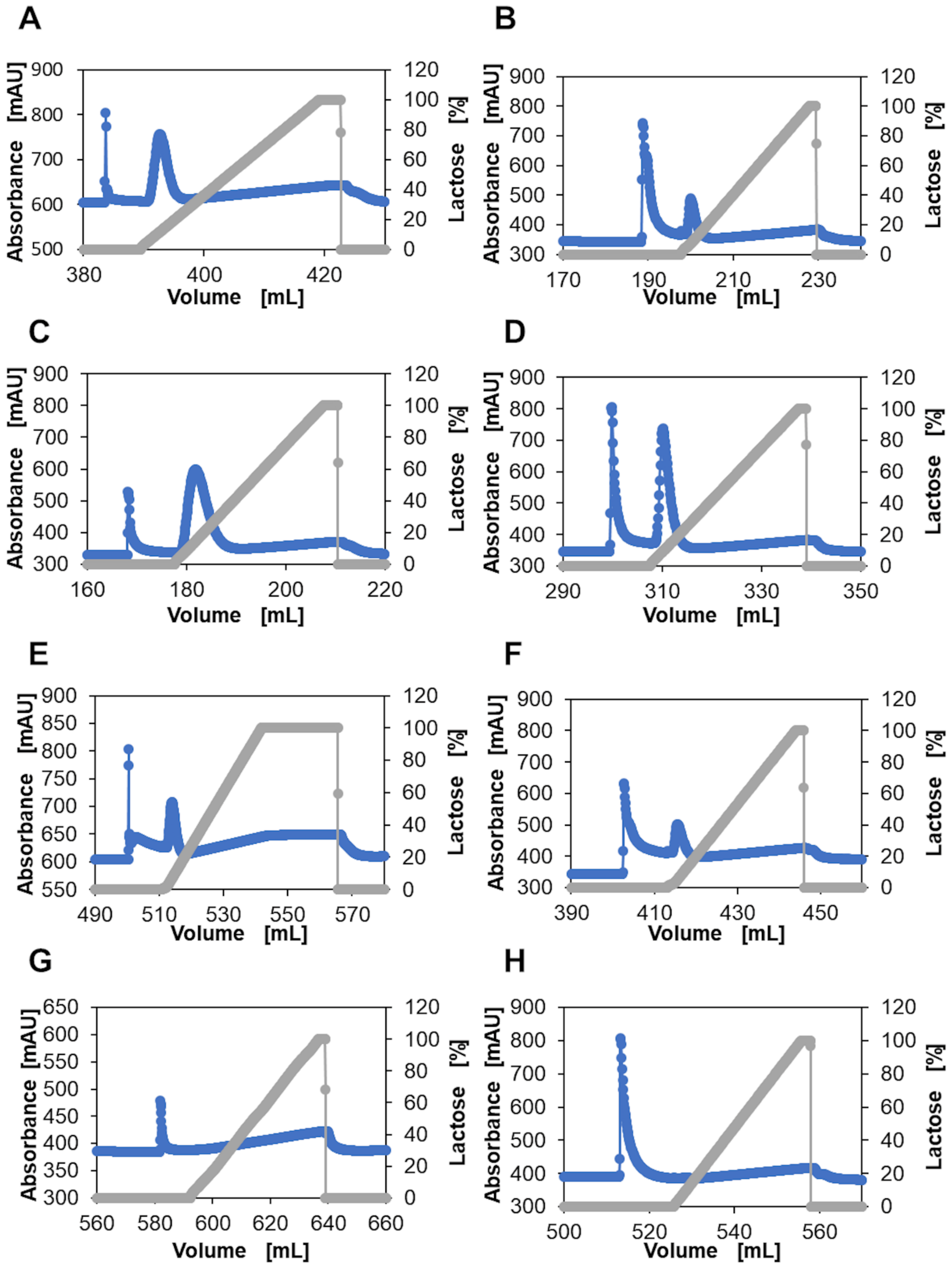


**Figure S4.** Elution profiles of galectin purification on ASF-Sepharose.

0.2 µmol galectin in PBS pH 7.5 was applied and eluted with lactose in a gradient mode (0-0.3 M lactose in PBS pH 7.5 within 15 min; 0%=0 mM lactose, 100%=0.3 M lactose). Blue line: Absorbance at 280 nm; Grey line: Concentration of lactose [%]. **A:** HGal-1C2S; **B:** HSDsRedMGal-1C2S; **C:** HGal-3; **D:** HSYGal-3; **E:** HGal-8N; **F:** HSeGFPGal-8N; **G:** HGal-8C; **H:** HSeGFPGal-8C.

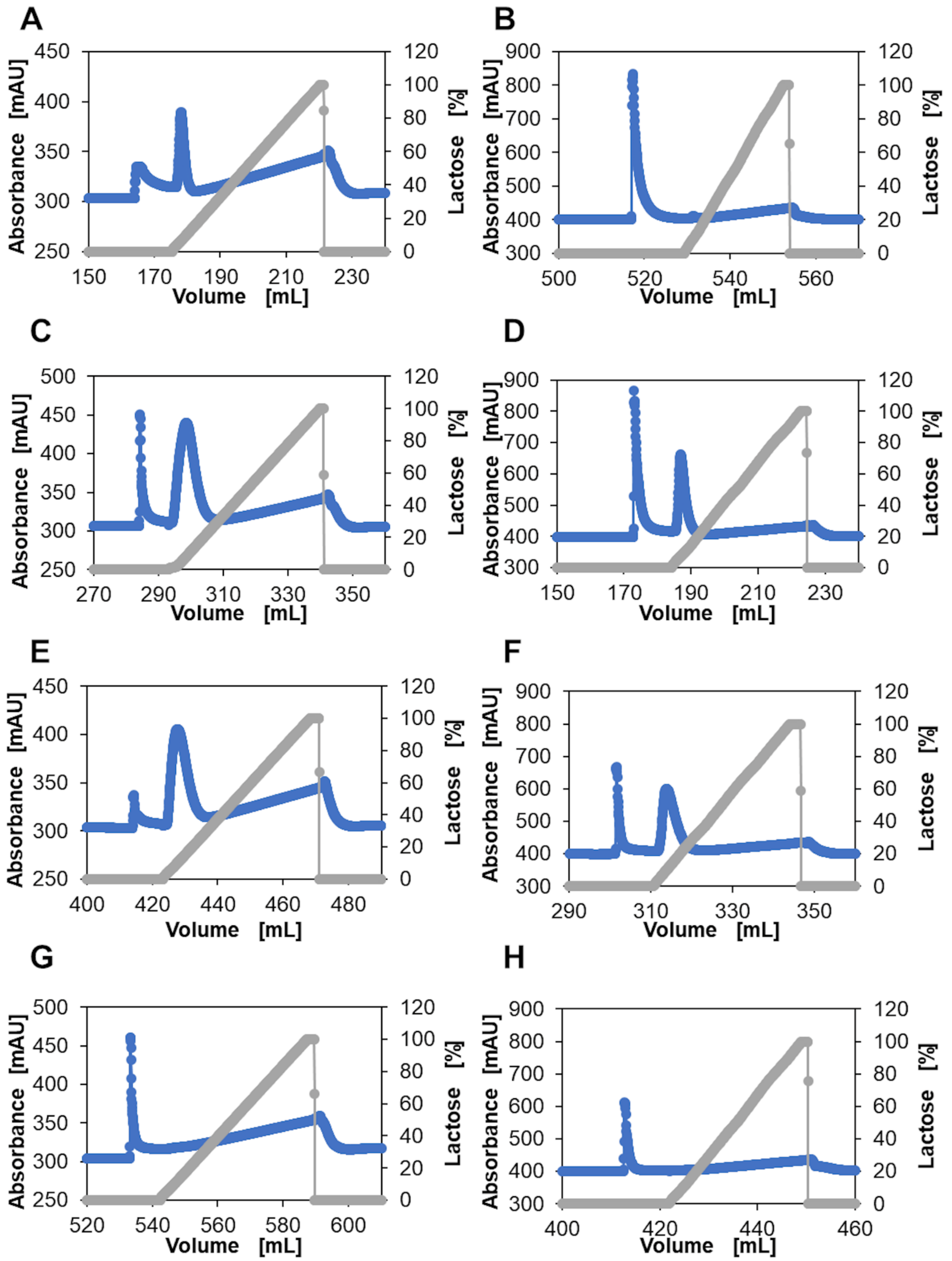


**Figure S5.** Elution profiles of galectin purification on Fetuin-Sepharose.
0.2 µmol galectin in PBS pH 7.5 was applied and eluted with lactose in a gradient mode (0-0.3 M lactose within 15 min; 0%=0 mM lactose, 100%=0.3 M lactose). Blue line: Absorbance at 280 nm; Grey line: Concentration of lactose [%]. **A:** HGal-1C2S; **B:** HSDsRedMGal-1C2S; **C:** HGal-3; **D:** HSYGal-3; **E:** HGal-8N; **F:** HSeGFPGal-8N; **G:** HGal-8C; **H:** HSeGFPGal-8C.

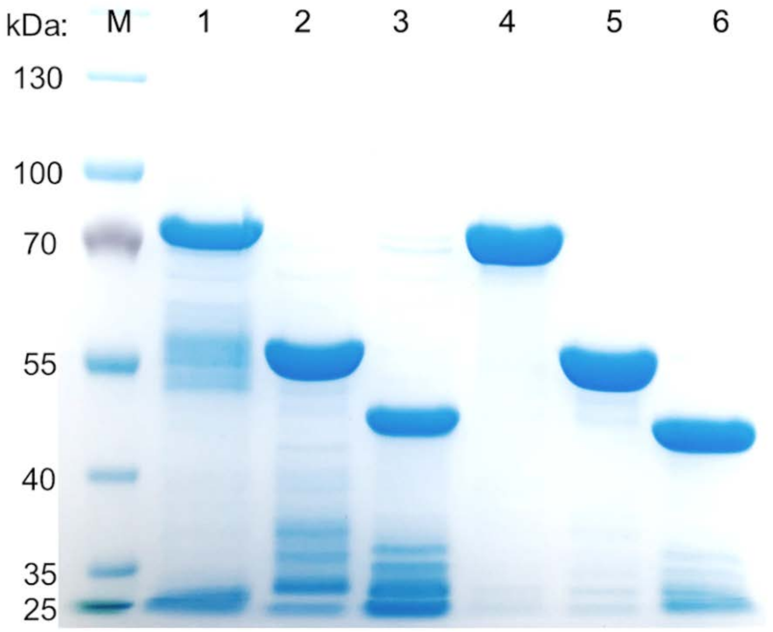


**Figure S6.** SDS-PAGE of Gal-3 fusion proteins after IMAC and ASF affinity chromatography.
M: Protein-Standard (10-180 kDa); **1:** HSYGal-3 IMAC eluate (74.2 kDa); **2:** HYGal-3 IMAC eluate (56 kDa); **3:** HSGal-3 IMAC eluate (46.9 kDa); **4:** HSYGal-3 ASF affinity resin eluate (74.2 kDa); **5:** HYGal-3 ASF affinity resin eluate (56 kDa); **6:** HSGal-3 ASF affinity resin eluate (46.9 kDa).

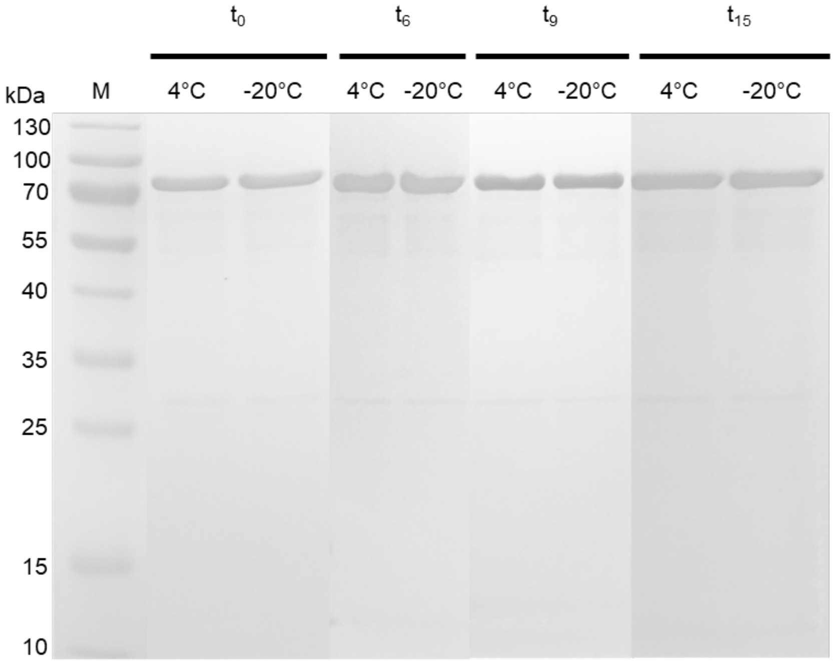


**Figure S7.** Analysis HSYGal-3 storage stability.
HSYGal-3 was purified with IMAC and ASF-Sepharose and stored at 4°C and -20°C in EPBS pH 7.5. Samples were taken on different days after purification ($t_0$, $t_6$, $t_9$, $t_{15}$) and in each case 5 µg protein was analyzed using discontinuous SDS-PAGE.

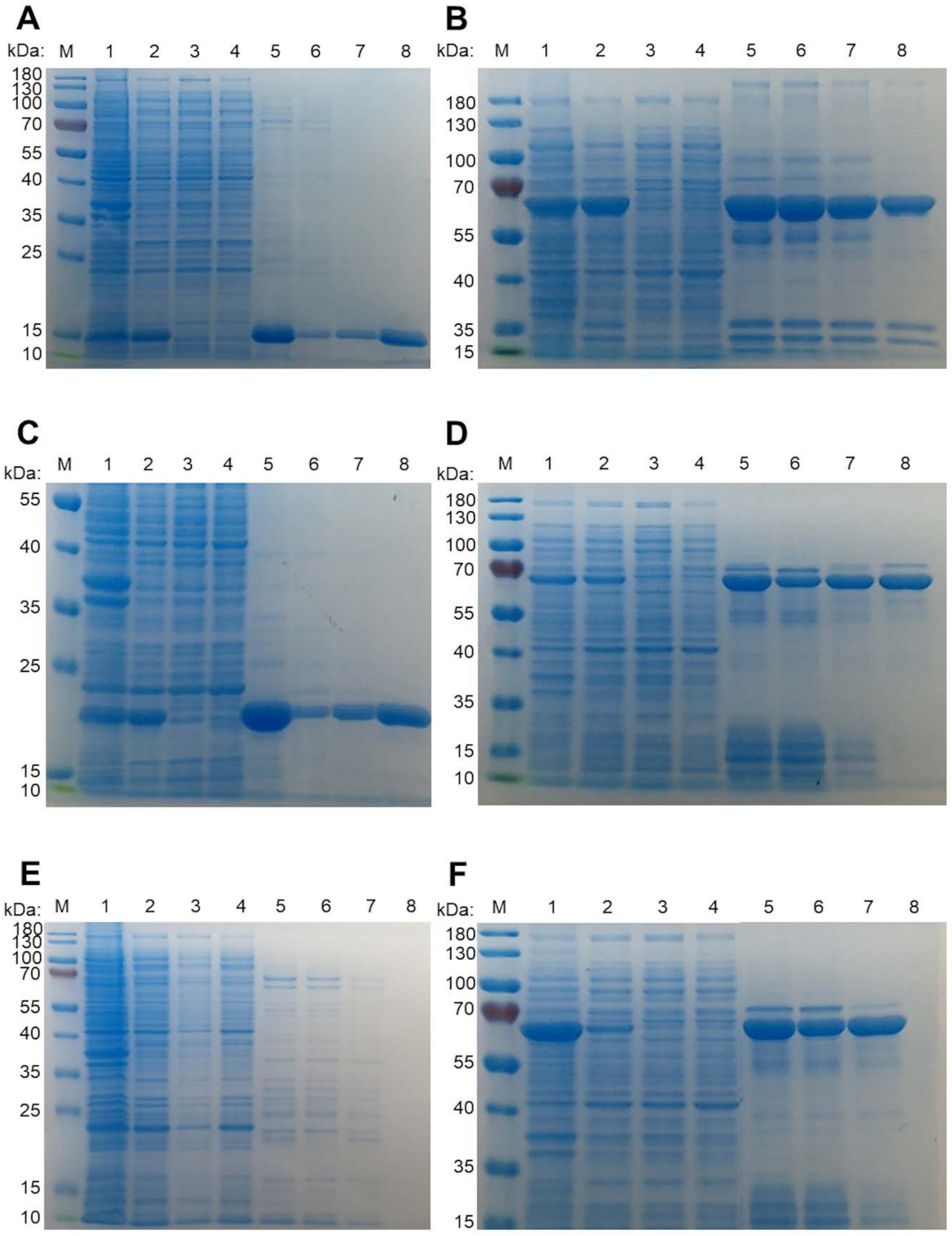


**Figure S8.** SDS-PAGE analysis of IMAC and ASF glycoprotein resin purified galectins**.**
**A:** HGal-1C2S (16 kDa); **B:** HSDsRedMGal-1C2S (64 kDa); **C:** HGal-8N (20.4 kDa); **D:** HSeGFPGal-8N (66.4 kDa), **E:** HGal-8C (17.8 kDa); **F:** HSeGFPGal-8C (63.9 kDa); M. Protein Standard (10-180 kDa); 1: Pellet; 2: Crude extract: 3: IMAC Flowthrough; 4: IMAC wash step; 5: IMAC eluate; 6: ASF affinity resin flowthrough; 7: ASF affinity resin wash; 8: ASF affinity resin eluate.

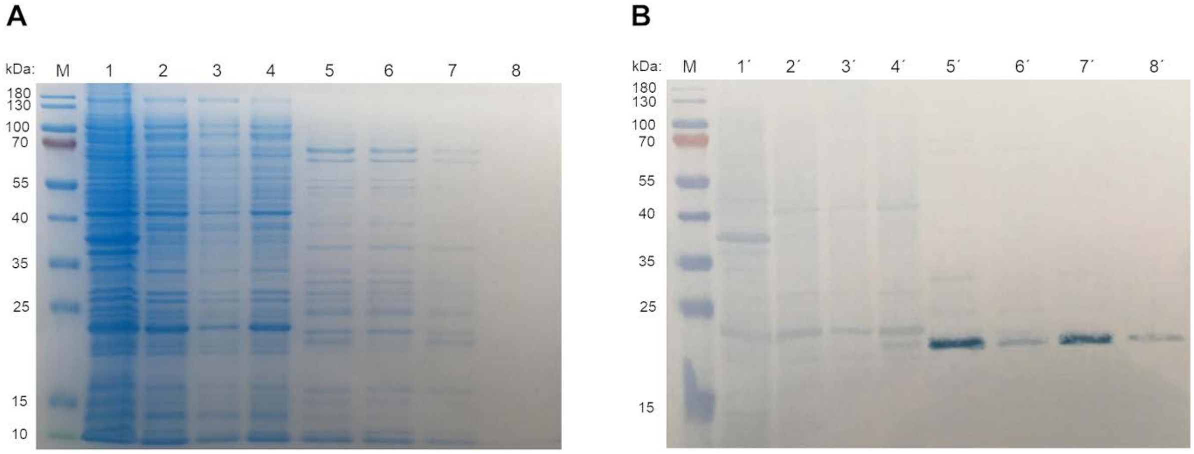


**Figure S9.** SDS-PAGE (**A**) and Western blot (**B**) of IMAC and ASF-affinity purification of HGal-8C.
**A:** SDS-PAGE with Coomassie staining: M. Protein Standard (10-180 kDa); 1: Pellet; 2: Crude extract: 3: IMAC Flowthrough; 4: IMAC wash step; 5: IMAC eluate; 6: ASF affinity resin flowthrough; 7: ASF affinity resin wash; 8: ASF affinity resin eluate. **B:** Western blot analysis with anti-$His_6$-antibody: M. Protein Standard (10-180 kDa); 1´: Pellet; 2´: Crude extract: 3´: IMAC Flowthrough; 4´: IMAC wash step; 5´: IMAC eluate; 6´: ASF affinity resin flowthrough; 7´: ASF affinity resin wash; 8´: ASF affinity resin eluate; $M_W$ (HGal-8C) =17.8 kDa.

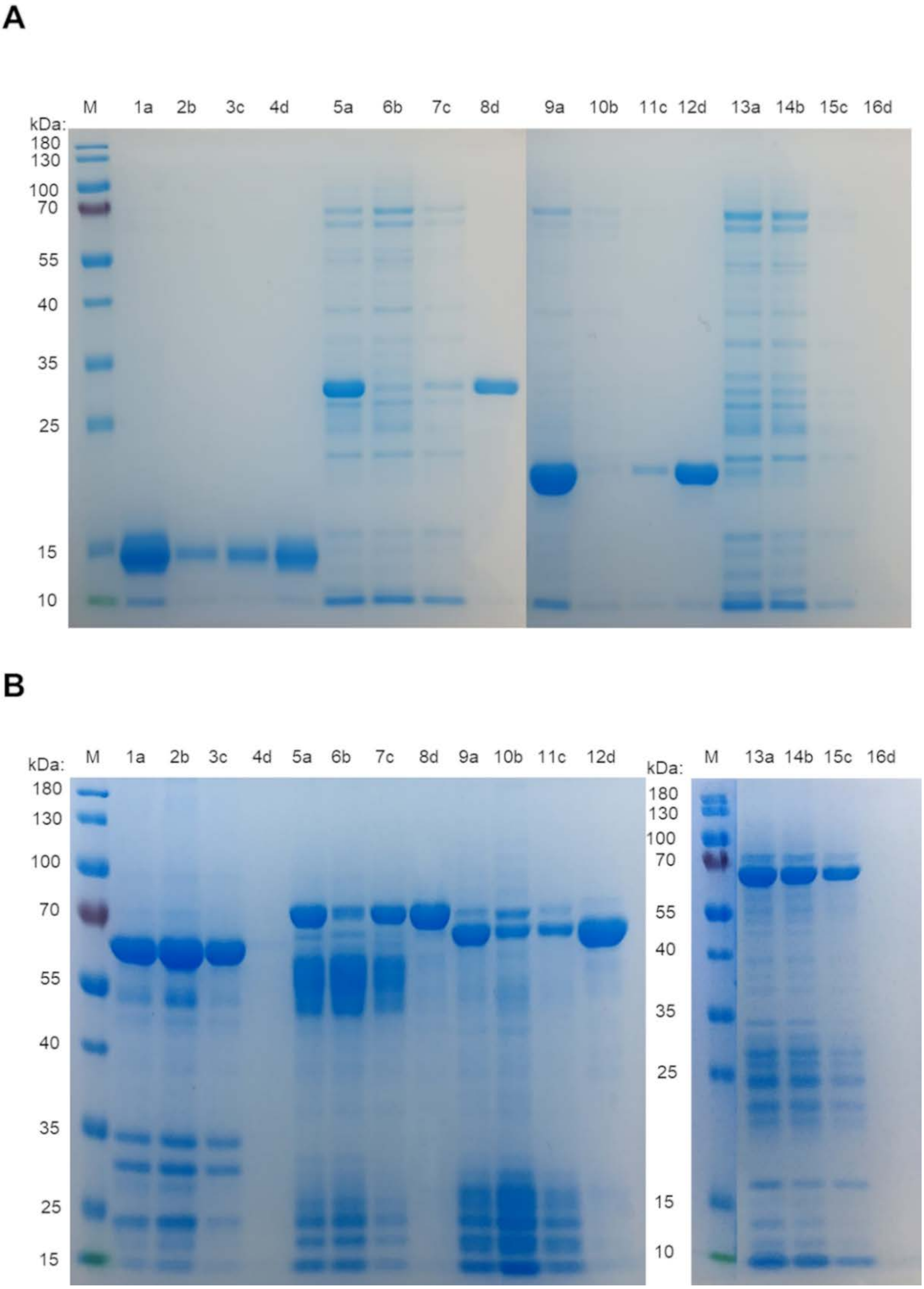


**Figure S10.** SDS-PAGE analysis of IMAC and fetuin affinity purified galectins.

**A:** $His_6$-tagged galectins (1-4:HGal-1C2S (16 kDa), 5-8 HGal-3 (28 kDa), 9-12: HGal-8N (20.4 kDa), 13-16 HGal-8C (17.8 kDa)); **B:** Galectin fusion proteins (1-4: HSDsRedMGal-1C2S (64 kDa), 5-8 HSYGal-3 (74 kDa), 9-12: HSeGFPGal-8N (66.4 kDa), 13-16 HSeGFPGal-8C (63.9 kDa)); for each galectin from left to right: a: IMAC eluate, b: fetuin affinity resin flowthrough, c: fetuin affinity resin wash, d: fetuin affinity resin eluate; M: Protein standard (10-180 kDa).

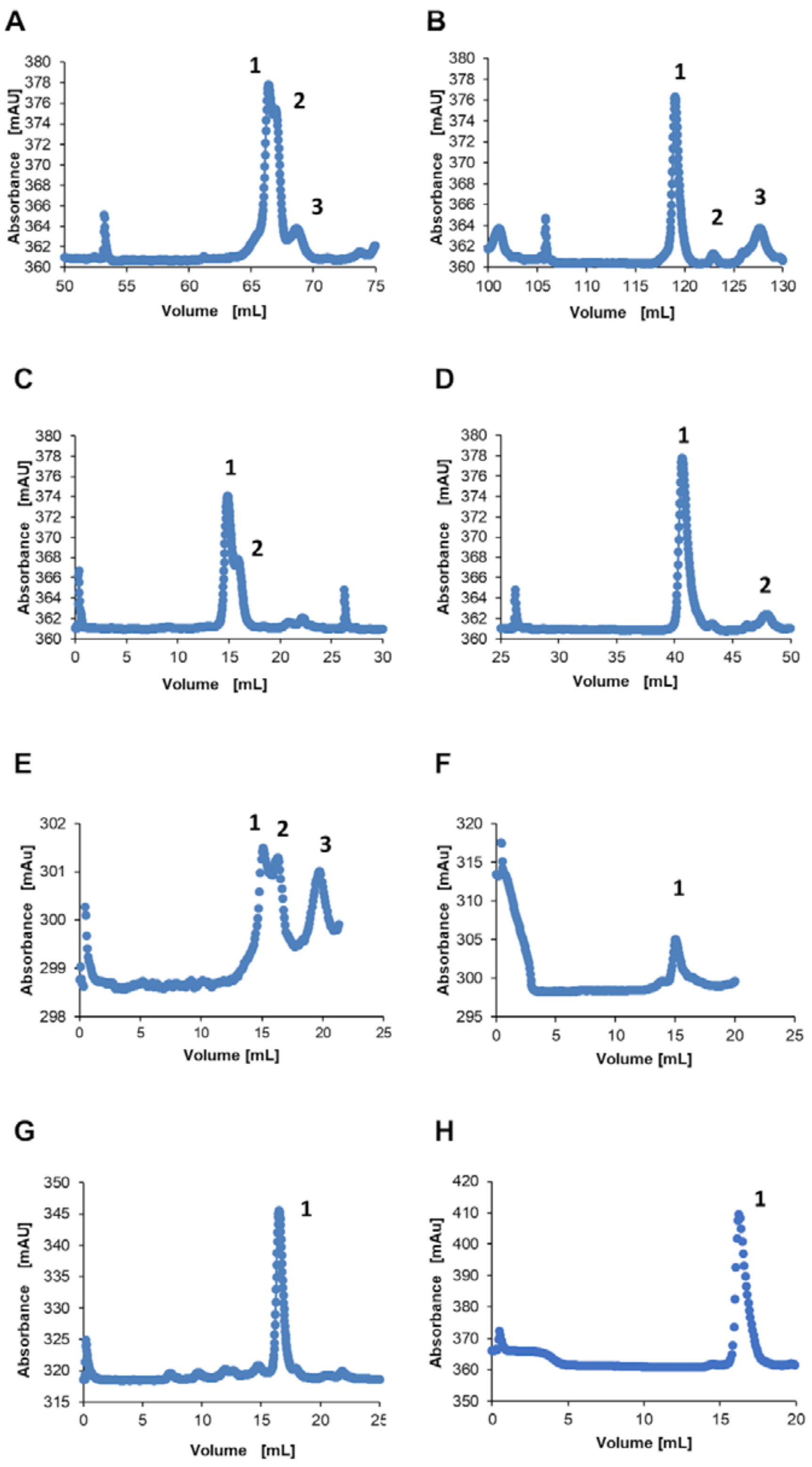


**Figure S11.** SEC profiles of Gal-3 proteins.
**A:** HSYGal-3 IMAC **B:** HSYGal-3 IMAC + ASF resin; **C:** HYGal-3 IMAC ; **D:** HYGal-3 IMAC + ASF resin resin; **E:** HSGal-3 IMAC; **F:** HSGal-3 IMAC + ASF resin resin; **G:** HGal-3 IMAC; **H:** HGal-3 IMAC + ASF resin resin. Column: Superdex 200 Increase 10/300 GL; Flow: 0.75 mL*min$^{-1}$; Buffer: PBS pH 7.5 Injection volume: 200 µL; Sample amount: 0.0024 µmol; $M_W$ (theoretic for monomers): HSYGal-3: 74.2 kDa, HYGal-3: 56 kDa, HSGal-3: 46.9 kDa, HGal-3: 28 kDa.

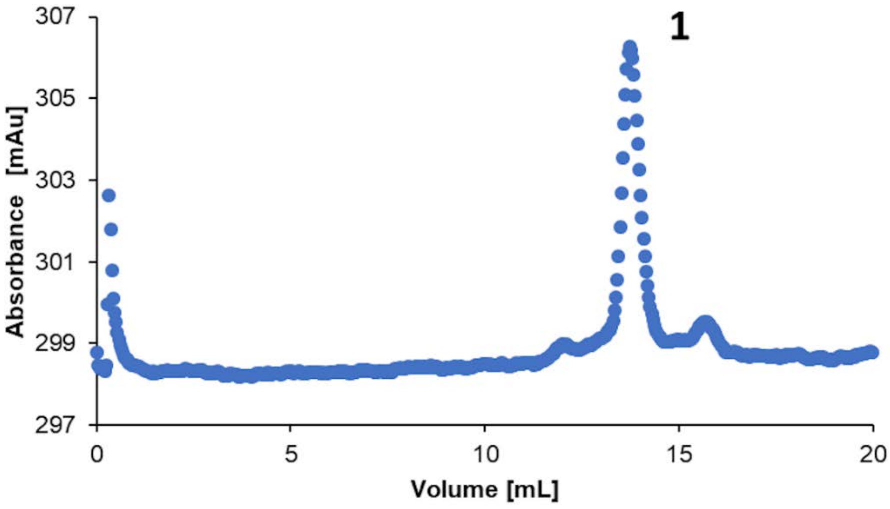


**Figure S12.** SEC profile of HSY eluate after IMAC.
Column: Superdex 200 Increase 10/300 GL; Flow: 0.75 mL*min$^{-1}$; Buffer: PBS pH 7.5 Injection volume: 200 µL; Sample amount: 0.0024 µmol; Peak 1: Elution volume: 13.73 mL, $MW_{(calc.)}$: 88.9 kDa; $M_W$ (HSY-Monomer): 48.1 kDa.

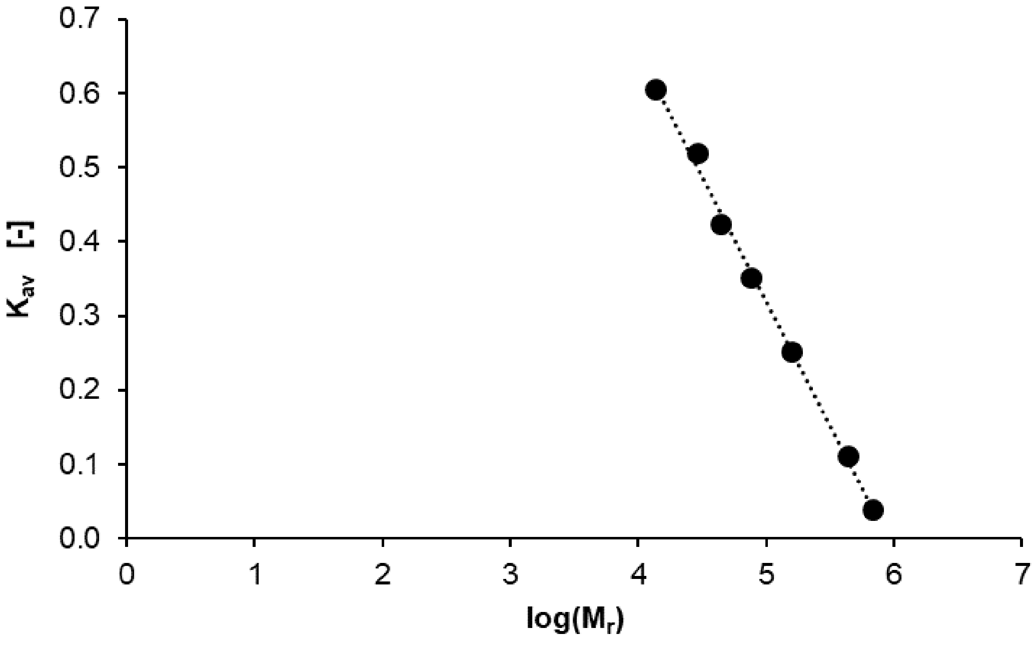


**Figure S13.** Linear regression of protein standards in SEC.
$K_{av}$ is plotted over the logarithm of the molecular weight ($M_r$). Molecular weights of detected samples in SEC were calculated by equation y=-0.335x + 1.936 ($R^2$=0.997). Protein standards: Thyroglobulin (669 kDa), Ferritin (440 kDa), Aldolase (158 kDa), Conalbumin (75 kDa), Ovalbumin (44 kDa), Carbonic anhydrase (29 kDa), Ribonuclease A (13.7 kDa).

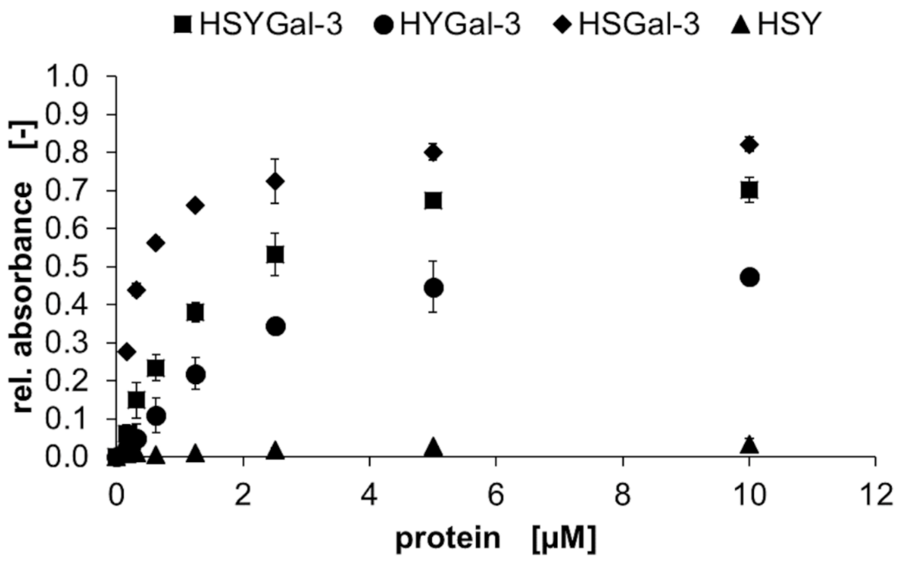


**Figure S14.** Binding of Gal-3 fusion proteins after IMAC.
The binding of IMAC-purified proteins HSYGal-3 (■), HSGal-3 (◆), HYGal-3 (●), and HSY (▲) (control) to ASF is shown. Bound galectins were detected using a peroxidase-conjugated anti-$His_6$-antibody and conversion of TMB as the mean signal of three data points. Errors indicate standard deviations.

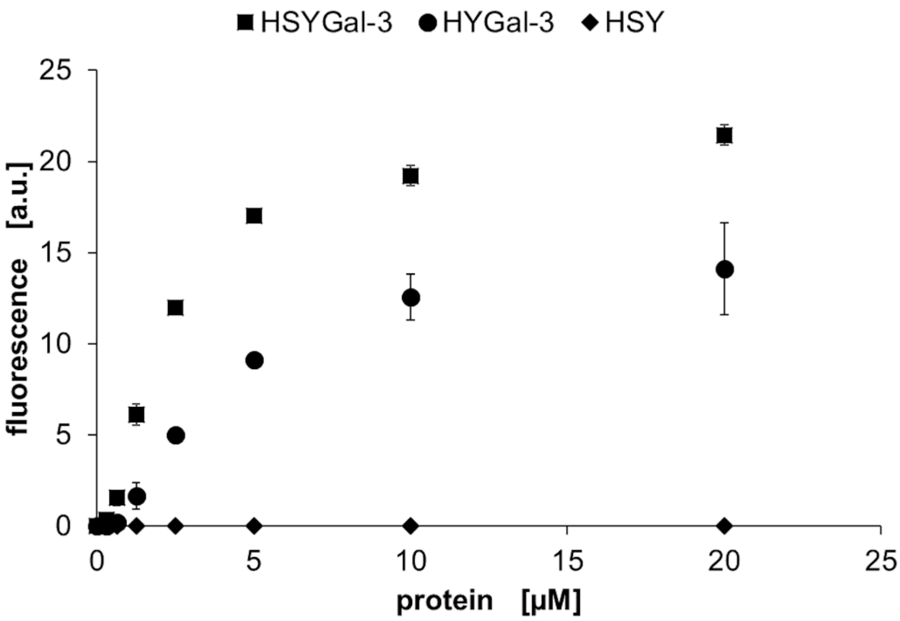


**Figure S15.** Binding of Gal-3 fusion proteins with fluorescence detection.
Binding of IMAC + ASF-Sepharose purified HSYGal-3 (■), HYGal-3 (●), and HSY (control, only IMAC) (◆) to ASF in a solid-phase binding assay. Fluorescence intensity was measured using excitation filter λ=485/20 and emission filter λ=528/20 as the mean signal of three data points. Error bars indicate standard deviations.

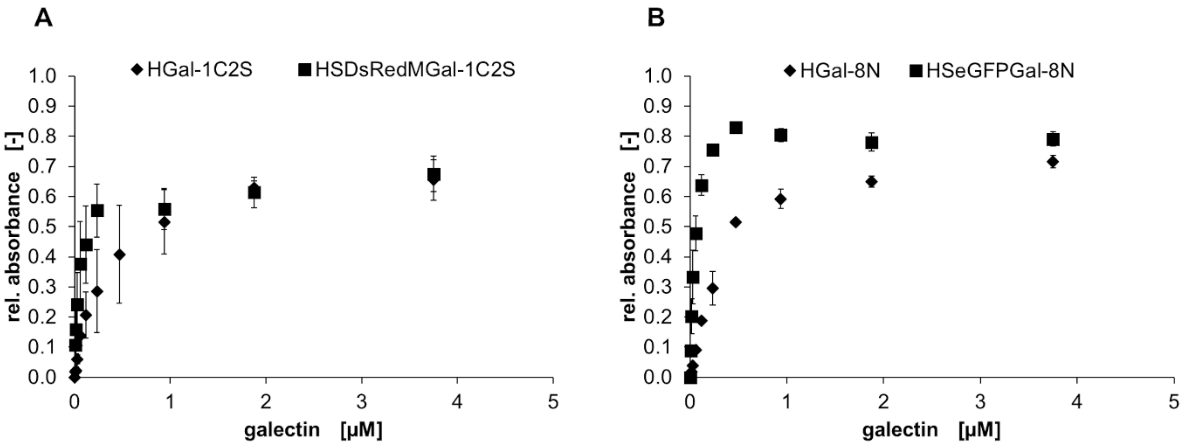


**Figure S16.** Binding of His$_6$-tagged and fusion proteins of Gal-1C2S and Gal-8N.

The binding of His$_6$-tagged proteins (◆) and fusion proteins (■) to ASF is shown for Gal-1C2S (**A**) and Gal-8N (**B**) in a solid-phase binding assay. Galectin binding was detected using a peroxidase-conjugated anti-His$_6$-antibody and conversion of TMB as the mean signal of three data points. Errors indicate standard deviations.

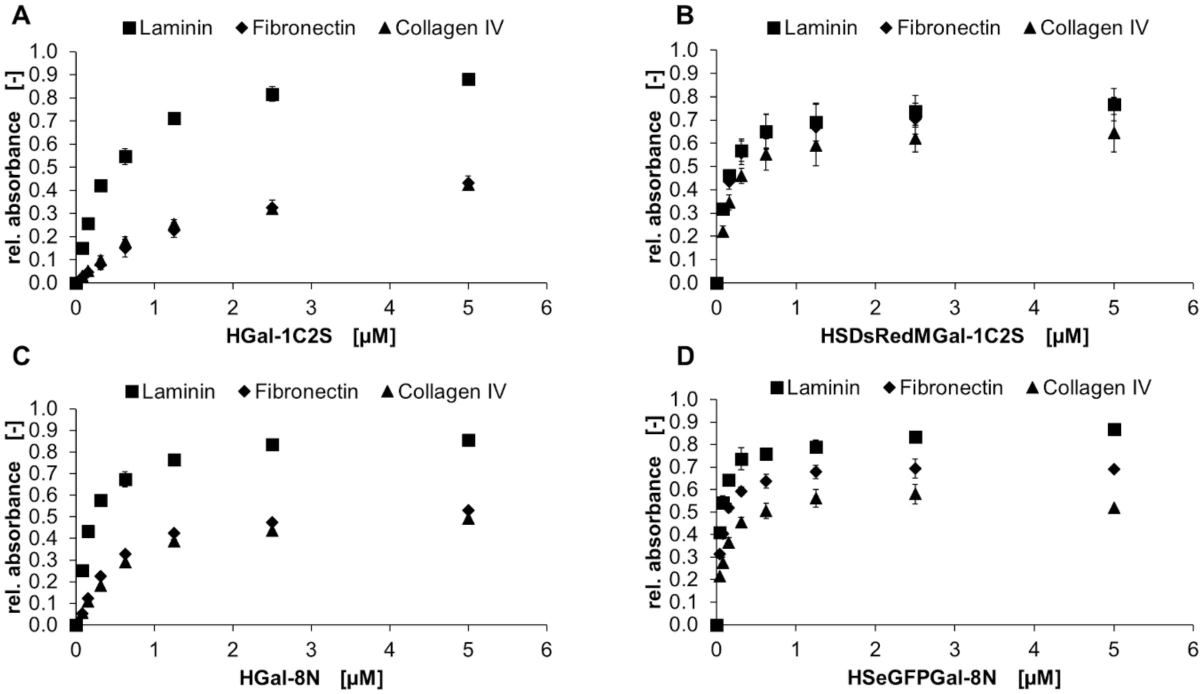


**Figure S17.** Binding of His$_6$-tagged and galectin fusion proteins on ECM glycoproteins.

The binding of galectins to laminin (■), fibronectin (◆), and collagen IV (▲) in a solid-phase assay is shown. **A:** HGal-1C2S; **B:** HSDsRedMGal-1C2S; **C:** HGal-8N; **D:** HSeGFPGal-8N. Galectin binding was detected using a peroxidase-conjugated anti-His$_6$-antibody and conversion of TMB as the mean signal of three data points. Errors indicate standard deviations.

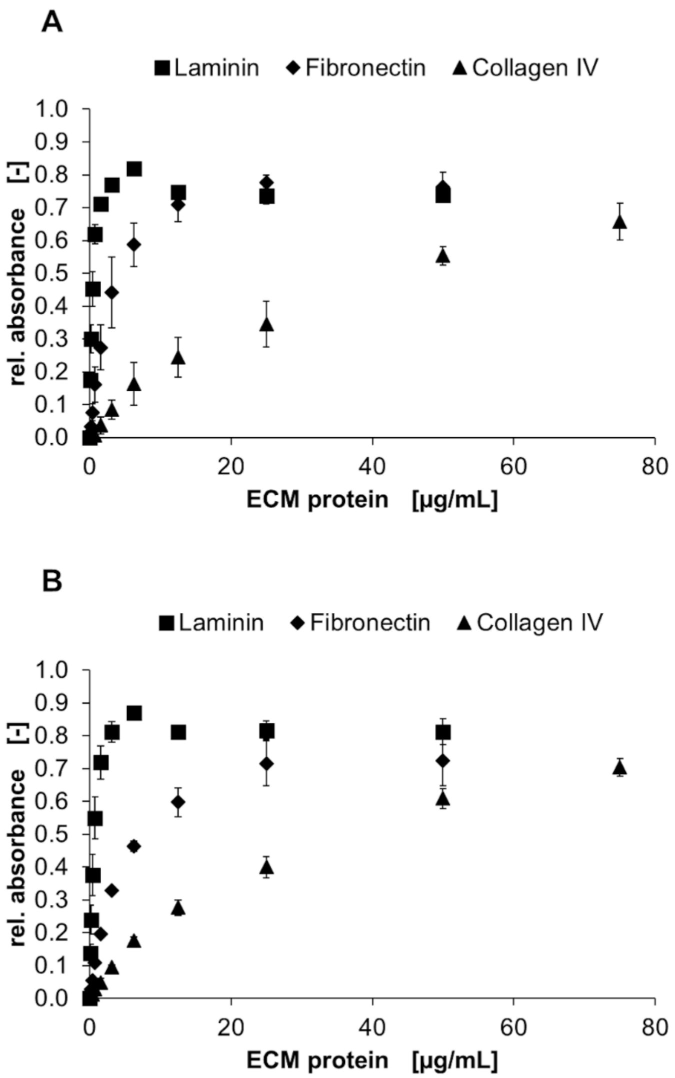


**Figure S18.** Binding of ECM glycoproteins to ASF-bound HGal-3 and HSYGal-3.
Binding curves of HGal-3 (**A**) and HSYGal-3 (**B**) for crosslinking laminin (■), fibronectin (◆), and collagen IV (▲) are shown. ECM glycoprotein binding was detected by primary anti-ECM protein-antibodies and corresponding secondary peroxidase-conjugated antibodies. Conversion of TMB was measured using three data points. Error bars indicate standard deviations.

**Table S3.** Calculated apparent $K_D$ values of laminin, fibronectin, and collagen IV binding to ASF-bound HGal-3 and HSYGal-3 in a solid-phase binding assay. Values were calculated by non-linear regression assuming one-site saturation.

| | **Apparent $K_D$ [µM]** | | |
|---|---|---|---|
| | **Laminin** | **Fibronectin** | **Collagen IV** |
| HGal-3 | 0.27 ± 0.03 | 5.1 ± 0.1 | 41.4 ± 8.5 |
| HSYGal-3 | 0.45 ± 0.04 | 10.0 ± 1.1 | 34.7 ± 3.1 |

**Table S4.** *E.coli* strains used for recombinant expression of galectin constructs.

| ***E.coli* strain** | **Galectin construct** |
|---|---|
| BL21 (DE3) | pET28a::HYGal-3 |
| Rosetta (DE3) pLysS | pET28a::HDsRedMGal-1C2S<br>pETDuet-1:: HGal-1C2S<br>pETDuet-1:: HGal-3<br>pETDuet-1:: HGal-8N<br>pETDuet-1:: HGal-8C<br>pET17b::HSDsRedMGal-1C2S<br>pET17b::HSYGal-3<br>pET17b::HSeGFPGal-8N<br>pET17b::HSeGFPGal-8C<br>pET17b::HSGal-3<br>pET17b::HSY |
| Rosetta 2 (DE3) pLysS | pET28a::HeGFPGal-8N<br>pET17b::HSGal-8N<br>pET17b::HSGal-1C2S<br>pET17b::HSDsRedM<br>pET17b::HSeGFP |
